# Experiences of women facing intimate partner violence during the revocation of protective orders^*^

**DOI:** 10.1590/1980-220X-REEUSP-2025-0010en

**Published:** 2025-12-05

**Authors:** Paula Sales Rodrigues, Juliana Ribeiro da Silva Vernasque, Aline Pereira de Souza, Viviane Boacnin Yoneda Sponchiado, Fabiana Veronez Martelato Gimenez, Maria José Sanches Marin

**Affiliations:** 1 Faculdade de Medicina de Marília, Marília, SP, Brazil.; 2 Faculdade de Medicina de Botucatu, Programa de Pós-graduação de Doutorado em Enfermagem, Botucatu, SP, Brazil.; 3 Polícia Civil do Estado de São Paulo, Delegacia de Defesa da Mulher, Marília, SP, Brazil.

**Keywords:** Domestic Violence, Violence Against Women, Intimate Partner Violence, Gender Rights, Grounded Theory

## Abstract

**Objective:**

To interpret the lived experiences of women in situation of violence who request the revocation of emergency protective orders.

**Method:**

Strauss and Corbin’s Grounded Theory (GT) and Symbolic Interactionism were used at the Women’s Police Station in a town in the State of São Paulo, through interviews with 28 participants from two sample groups: women experiencing violence and police station professionals.

**Results:**

The results were organized into categories related to the components of the theoretical model, “*Experiencing the Request to revoke an Emergency Protective Order*,” highlighting the emotional and contextual challenges faced by women seeking formal help. These challenges are permeated by ambivalent feelings, experiences of revictimization, and different coping strategies to reframe their life trajectories.

**Final Considerations:**

The revocation is related to family consequences - guilt, regret, and sadness -, despite acknowledging the violence. The lack of understanding of the judicial system and the emotional bond with the perpetrator demonstrate the need to consider the symbolic meanings of interactions in primary care nursing practice.

## INTRODUCTION

Leaders from different countries around the world have outlined significant strategies for sustainable development, carrying out actions aiming at promoting equity in various contexts, in accordance with human rights and dignity^(1)^. From this perspective, for the first time in history, a global development agenda invests in the urgency of gender equality emphasized through Goal 5 of the Sustainable Development Goals (SDGs), with implications in various dimensions, be it in education, economy, health, harmful cultural practices, such as forced marriages, and the eradication of all forms of violence against women and girls^(2)^.

Notably, the intimate partner emerges as the main perpetrator of violence^(3)^. Globally, it is estimated that between 38% and 50% of homicides of women occur at the hands of intimate partners^(4)^, resulting from an ongoing pattern of abuse, lacking a more meaningful understanding of the contexts and motivations that influence the decision to remain in or break off a violent relationship^(5)^.

Brazil ranks fifth in the world in feminicides, which means that, on average, a woman is killed every four hours, totaling a daily rate of 4.3 female homicides per 100,000 women, almost double the global average^(6)^. This reality is more pronounced in unfavorable socioeconomic contexts, often invisible and stigmatized. Therefore, efforts to change the circumstances these women face are still insufficient^(7)^.

Intimate partner violence (IPV) is a phenomenon that affects both men and women. However, this study focuses on intimate partner violence committed by men against women, specifically those in common-law marriage or cohabiting, cisgender, heterosexual, and of female biological sex. It is important to emphasize that in Latin America and the Caribbean (LAC), including Brazil, discriminatory discourses and seemingly protective stances, expressed through hostile or benevolent sexism, reinforce normative gender stereotypes that restrict women’s autonomy^(8,9)^.

Often, after a complaint is filed criminally qualifying the perpetrator under the Maria da Penha Law (2006), an emergency protective order is instituted, preventing the perpetrator from maintaining contact with or approaching the woman. The order can only be revoked judicially, even if there is any request for its revocation^(10)^.

It is noteworthy that most interventions focus solely on the safety of women in situations of domestic violence, without considering the full range of consequences and complexities arising from these families’ ongoing contact with perpetrators. These complexities arise from interactional dynamics, the progressive and cyclical nature of both attempted breakups and parental relationships, essential elements for developing effective interventions that impact the experience of families seeking formal help^(7,10,11)^.

Furthermore, the phenomenon of IPV and the subsequent revocation of protective orders constitute a complex problem^(10)^ that demands an interdisciplinary approach. Therefore, the health sector must expand its understanding of the issue and strengthen its role in preventing the problem, as well as in intervention and health promotion. Primary health care (PHC), through health units, is the closest and most potential care reference for women experiencing violence. Nursing visits can play a strategic role in responding to IPV by promoting prevention and intervention based on longitudinal family care^(12)^.

The study seeks to contribute to advancements in the field of care by highlighting nuances often overlooked in traditional analyses of revocations of emergency protective orders by women receiving specialized services. From this perspective, this research seeks to interpret the experiences of women in situations of violence, who request the revocation of their emergency protective orders, through the research question: what are the experiences of women who request the revocation of their protective orders?

## METHOD

### Study Design

This qualitative study used Grounded Theory (GT) proposed by Strauss and Corbin^(13)^ as its method, and Symbolic Interactionism (SI)^(14)^ as its theoretical framework. GT (which is based on SI) consists of an inductive-deductive method aimed at interpreting the experiences of people inserted in a given social context and values the creation of theories based on the phenomenon under study, which is expressed by the paradigmatic model. SI considers social reality constructed by symbolic interaction among individuals, in which meanings are attributed to things and events based on social interactions, motivating their actions and interpretations of the world^(14)^.

The study was conducted following the assumptions of the Consolidated Criteria for Reporting Qualitative Research (COREQ)^(15)^.

### Study Location

The location was the *Departamento de Polícia Judiciária de São Paulo Interior* (DEINTER 4) in one of its sections, which included a Women’s Police Station (WPS), located in a medium-sized town in the Center-Western region of São Paulo, Brazil.

### Participants and Selection Criteria

The study involved women over 18 years in situation of violence with a registered police report who came to the Women’s Police Station to request the revocation of the emergency protective order (EPO) between September 2021 and July 2022. Gender expression was not considered an inclusion or exclusion criterion, implicitly assuming female gender expression. Those who demonstrated any impossibility to provide information were excluded. The first contact was made by police station professionals who, upon providing assistance, informed the interviewer about the study and, if interested and available, the researcher - a doctoral student with extensive professional experience in women’s health in primary care and in qualitative interview techniques was called upon. The researcher approached the women, addressed their needs, and explained the guidelines for participation in the study. A strategy of weekly on-call sessions on Mondays and Fridays was used to maximize recruitment. The women were progressively selected until no new information was available to understand the phenomenon at hand, as determined by the authors’ consent.

According to the theoretical sampling defined by the GT^(13)^, concepts that led the researchers to new questions and the development of hypotheses were raised, which, in turn, were answered in the new data collection. Therefore, this new process occurred concurrently with data collection and analysis, supported by the memoranda. It was noted that, on the one hand, the women felt embarrassed to return to the police station to request revocation; on the other hand, the strong involvement of the police station professionals was observed in an attempt to support these women, highlighting the consequences of returning to live with the perpetrator. Thus, the second sample group was established, composed of eight professionals, that is, all of them working at the Women’s Police Station (WPS), with no refusals.

### Data Collection and Organization

To adapt the semi-structured interview script, three pilot interviews were conducted during home visits and at the police station, resulting in minor adaptations. In the first sample group, 20 interviews were conducted, following the semi-structured script with questions about the repercussions of the protective order, the decision to revoke it, and the meanings and feelings involved. During the interviews, new questions were added, including a request for participants to fully describe the event that led to the request for the protective order. The strategy considered that, at the time of revocation, the women expressed in ways that differed from the initial content of the police report, influenced by multiple emotional, social, and contextual factors.

The interviews lasted an average of 40 minutes and were arranged via telephone to determine the interviewees’ preferred location and time, considering the privacy and security of both the interviewee and the researcher. Thus, four interviews were conducted at the interviewee’s home, once ensured their partners would not be present, and the remaining 16 took place in a private room at the police station.

For the second sample group, eight scheduled interviews were conducted at the workplace, following a semi-structured script containing, in addition to data on identification, questions about how they perceive the situation of women who request the revocation of emergency protective orders during their visits to the police station. These interviews lasted an average of 30 minutes, were audio-recorded using an MP3 player, and were conducted and transcribed verbatim by the lead researcher, a doctoral student with professional experience in women’s health and qualitative interview techniques. Transcription occurred immediately after each interview, initiating the analysis process to identify the need for further data analysis, as proposed by the GT.

### Data Analysis

According to the Straussian Grounded Theory (GT) method^(13)^, the analysis began with open coding. In this phase, the data were examined in detail, line by line, through constant questioning, aiming to conceptualize the meanings expressed by the participants, allowing an understanding of what each piece of data represents. In the second stage, with axial coding, the data were regrouped according to their relationships and connections, developing more comprehensive explanations of the phenomenon under investigation. This process aims to enhance specificity and conceptual rigor through an inductive and deductive movement, enabling the identification of patterns and relationships within the data. In this stage, there was an integration between the categories, and the main point was the discovery of the central category or phenomenon. In selective coding, the most relevant categories that integrated the data into a more comprehensive theory were chosen^(13)^; the NVIVO Plus software, version 11, was used.

In this context, we have the paradigmatic model, encompassing the following components: conditions, actions-interactions, and consequences. Conditions refer to people’s reasons or explanations for why/how they respond to problematic situations. Actions/interactions are the meanings they attribute to the problematic events/situations they experience and how they manage/achieve their goals. Consequences result from actions/interactions^(14)^.

It should be noted that the entire data analysis process was initially conducted by the lead researcher and subsequently analyzed by one of the researchers with experience in the analysis technique. During this process, notes and reflections were taken that led to a consensus on the codes, categories, and subcategories. Data interpretation was validated through feedback to the women and staff at the police station, as well as to the group of researchers involved, with the presentation of the results and reflections on the interrelationships that culminated in the theoretical model.

### Ethical Aspects

This study is part of the Research Project – “Domestic Violence Against Women: Experiences and Repercussions of Requests for Revocation of Emergency Protective Orders” – approved by the Research Ethics Committee (CEP) of the proposing institution, under No. 4,735,433. Informed Consent was obtained from all participants in the study, in writing, in person. In addition to ethical precepts, the names of the interviewees were replaced when referring to their statements in the text, with W (Woman) and P (Police Station Professional), followed by the number corresponding to the order in which the interviews were conducted.

## RESULTS

In characterizing the 20 women experiencing violence, we identified a prevalence of women aged 36 to 45, self-reported race (brown or black), and completed high school. Regarding the professionals, the majority were women, over 40, self-identified as white, who had been in service at the Women’s Police Station (WPS) between one and five years. The data analysis process yielded 1,192 substantive codes, 16 subcategories, and four categories. Chart 1 shows the distribution of the categories and their respective subcategories, culminating in the phenomenon: *“Experiencing the request to revoke an emergency protective order*.”

**Chart 1:** Distribution of Categories and Subcategories of the Phenomenon “Experiencing the request to revoke an emergency protective order” – Marília, SP, Brazil, 2025.

Phenomenon: *“Experiencing the request to revoke an emergency protective order”*		
Model component	Categories	Subcategories
Condition	Contextualizing the consequences of formalizing the complaint	• Pointing out consequences in children’s lives • Revealing consequences in the aggressor’s life • Feeling consequences in her own life • Feeling guilt, regret, fear and sadness
Action-complaint	Recognizing the cyclical dynamics of violence and its determinants	• Suffering different types of violence • Identifying behaviors that lead to violence • Experiencing violence repeatedly • Relating to the abusive use of alcohol and/or other drugs
Action-interaction	Justifying the revocation of the emergency protective order	• Believing that violence will no longer occur • Considering that it was not an assault • Ignoring the assumptions of the Maria da Penha Law • Experiencing impunity • Feeling love, affection and care
Consequence	Seeking ways to give new meaning to life	• Seeking to live independently from the aggressor • Identifying elements for an effective care network • Having faith, strength and hope

By the authors, 2025.

In the interpretation of the central phenomenon *“Experiencing the request to revoke an emergency protective order”,* the category “ Contextualizing the consequences by formalizing the complaint” is considered as *a condition; the actions-interactions* component was verified in the categories: “ Recognizing the cyclical dynamics of violence and its conditioning factors” and “Justifying the revocation of the emergency protective order”. The category: “Seeking ways to give new meaning to life” was defined as *consequence.*

In this context, it was possible to observe that, after filing a police report and requesting an emergency protective order, women experience repercussions in their own lives, in their children and in the life of the perpetrator, provoking a mix of emotions. In addition, the order brings up difficulties and needs that require confrontation, which is not always possible given the emotional and social resources available to them.

Despite the condition of requesting the revocation of the protective order, they acknowledge the existence of violence manifested in different forms, particularly physical aggression, psychological and moral violence, as well as the relentless repetition of these episodes. Furthermore, the motivations these women list for the violent events refer to the demand for separation, interconnected with the use of psychoactive substances, such as alcohol and/or illicit drugs. However, in this movement of action and interaction, they seek to justify the request for revocation of the emergency protective order, suggesting an oscillation between the desire for protection and regret for their decisions. Consequently, the intention to overcome this condition through the pursuit of independence from the perpetrator and attachment to religion is evident.

Interpretations of the phenomenon are also supported by observations made by Police Station professionals, who highlight the progressive severity of episodes of violence and the use of protective orders as a strategy for protection and, contradictorily, for power and control over the perpetrator.

**Figure 1:**
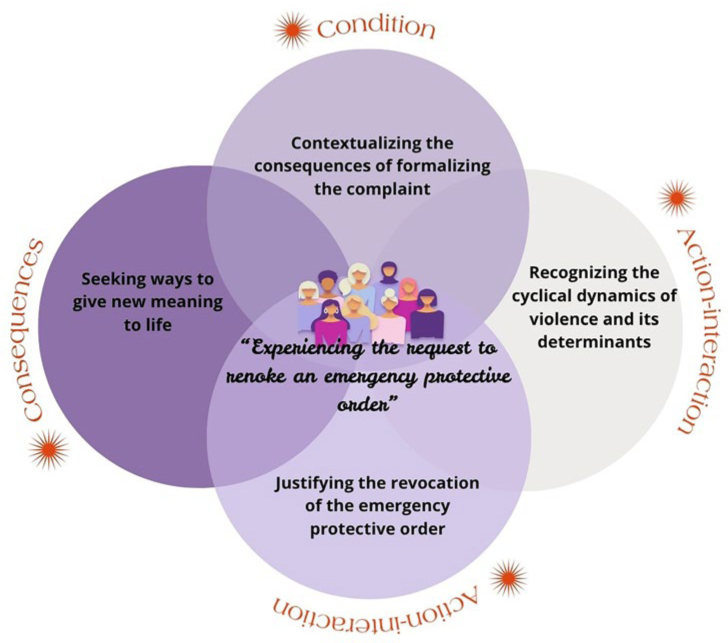
Phenomenon “Experiencing the request to revoke an emergency protective order”. Brazil, 2025.

The analysis allowed the elaboration of the paradigmatic model presented (Figure 1) based on the Grounded Theory. This construction is based on the interpretation of the central phenomenon - *“Experiencing the request to revoke an emergency protective order”* - as a cyclical and dynamic experiential process of decision-making, influenced and intertwined by social, emotional, and contextual dimensions.

### Condition - Contextualizing the Consequences of Formalizing the Complaint

Filing a police report and requesting an emergency protective order has consequences for the lives of these women, their children, and the perpetrators themselves. The children, despite wanting their mothers’ protection, experience the absence of their father and suffer as a result. The perpetrators exploit women’s expressed desire to return to family life, evidenced and reported through requests for reconciliation, fear of imprisonment, and the negative impact on their professional ties. For the women, filing police reports elicited emotional repercussions, described by a variety of feelings, including guilt, regret, fear, frustration, and shame for having filed a report and initiated judicial proceedings. For police station professionals, the increased attention is heightened due to the risk of coercion associated with the revocation of emergency protective orders.

*[...] my little one had a fever this week, she asked: mom, when is dad coming back.* (W4)

*[...] we are fine but very upset about what happened and afraid he will be arrested.* (W11)

*[...] he was desperate, he sent a lot of messages asking for forgiveness to come back home [...]* (W17)

*The fact that I had to come here to file a police report made me feel very embarrassed in front of people.* (W13)

*[...] when they return to revoke the order, I understand that they regretted it, often forgave them. [...] I believe that they would be coerced into requesting the revocation, but generally, they claim to be hot-headed and forgiven them.* (P3)

*[...] there is concern about whether or not she is in fact being coerced [...]* (P6)

### Action-Interaction - Recognizing the Cyclical Dynamics of Violence and Its Determinants

Despite requesting the revocation of the protective order, the interviewees acknowledge that they experienced different types of violence. Physical violence manifested itself in acts such as punches, kicks, beatings, and strangulation, revealing the viciousness and cruelty of the act. Patrimonial violence manifested in different forms was characterized by the destruction of belongings and property, resulting in losses. Psychological and moral violence were demonstrated by the perpetrator’s statements, which were laden with threats and emotional blackmail, and offensive, devaluing, and derogatory terms, even regarding physical appearance.

The reports highlight the dynamics of marital relationships based on recurrent violence and neglect of previous legal orders. For the Police professionals, there is a sense of progressive severity of the acts and an increase in cases with repeat patterns.

*He would turn my arm like that, with such malice, with such cruelty, that you could see in his eyes that he really wanted to break it.* (W6)

*Then he kept calling me a slut... you’re fat and ugly [...] if I left him, he would kill me.* (W1)

*[...]he doesn’t get aggressive with me, but he throws objects inside the house.* (W15)

*Because there was a protective order, but he never respected it[...]* (W19)

*[...] because what was an insult became a slap, a threat, a broken arm, a broken tooth. We say, the next one will be a police report of their death? But they don’t get shaken.* (P2)

*[...]domestic violence rarely improves, it usually gets worse.* (P6)

*In fact, she just said that at the beginning of the year, she had taken out a protective order and revoked it because she believed her husband’s promises.* (P7)

Women highlight factors that trigger violence, including behaviors such as jealousy, possessiveness, control over the victim, emotional instability, distrust, and requests for separation. There are reports of experiencing violence throughout their lives, both by women and perpetrators, including previous abusive relationships and childhood trauma. They strongly associate the abuse of alcohol and illicit drugs with violence, by the perpetrator or both, resulting in loss of control and aggressive behavior.

*[...]he threatened me that if I separated or asked for a divorce he would make my life hell, that he would go to my work, that he would take the car away [...]* (W12)

*He is jealous, very troubled [...] Comings and goings, fights, very possessive, he saw things [...]* (W10)

*[...]when he drinks, he has a macho side, a bad temper, he says: I’m really bad, I’m not good.* (W5)

*[...] he has a violent history [...]* W

*Each case is different [...] in many cases the victim had a childhood like this, for them it is normal.* (P8)

In this context, the women considered that the motivation for seeking the Police Station was the search for relief.

*[...] then I plucked up the courage and came to the police station.* (W4)

*[...] for me, the police station was a point of relief.* (W1)

*[...]you become useful again! An example, an instruction, of telling the person what they can do to improve that situation [...] It’s an experience that I will take with me for my life.* (P7)

### Action-Interaction - Justifying the Revocation of the Emergency Protective Order

Despite recognizing the brutality of violence and that its inherent factors often involve habits that are difficult to eliminate, the interviewed women justify their revocation based on the belief that the violence will not be repeated. There is a desire to rebuild the perpetrator’s image, whether by admitting guilt, alleging rashness, or even claiming they lied when reporting the matter. Furthermore, there is a desire to support the family, the perpetrator’s remorse, feelings of love, and emotional and financial dependence.

Police professionals, in turn, point out that the use of protective orders represents a way to exert power and control over the perpetrator’s actions. Furthermore, lack of knowledge about the Maria da Penha Law leads to the use of the EPO as a temporary and easily revoked form of protection.

*And it won’t happen again [...] he was very clear that he didn’t want to do that. He apologized [...]* (W2)

*I rushed it but I didn’t need to [...] we’re fine, going to church, if one day it doesn’t work out, I’ll break up and that’s it.* (W12)

*[...] the emotional bond is very strong.* (P4)

*I’m going to have to tell the truth, I lied even though I ran the risk of being sued [...]* (W11)

*[...]sometimes it’s the only way they have, given what they’re experiencing, to have power, control in their hands, a bargaining chip.* (P2)

*[...] she convinces herself that the person will change because she wants to convince herself, because she likes the person, so she thinks that by taking the order and then revoking it, it will solve the problem, and no, it doesn’t.* (P4)

### Consequences - Seeking Ways to Give New Meaning to Life

The interviewees revealed a desire to rebuild their lives, with or without the perpetrator, motivated by the search for financial independence and legal alternatives, such as pursuing civil separation proceedings. Some women mobilized emotional and spiritual resources to move forward with their partners, recognizing their own potential, the need for dialogue, and the need to distance themselves from “potentially harmful” family members.

Police professionals point to the need for an effective care network in cases of domestic violence, aiming to reduce revictimization and revoke protective orders.

*I want to work, submit my resume. [...] I have a lawyer and I’m going to separate.* (W1)

*[...] I want to go back to work, whether I’m with him or not.* (W4)

*I started taking the courses I wanted to take, living my life [...] thinking differently, seeing that I can do things without him.* (W3)

*[...] we are talking a lot, going to church and respecting each other.* (W12)

*[...]I believe that God will change his ways [...]that’s what has given me strength, my faith.* (W17)

*[...] they really need psychological treatment [...]* (P2)

*[...] we need to have a multidisciplinary team to provide the initial reception [...] health professionals, social workers, and the public defender’s office would be all that is needed.* (P4)

## DISCUSSION

Among the women in situations of domestic violence in this study, socioeconomic data, such as race, age, and education, reveal characteristics of full reproductive development and potential for economic and social advancement, which, paradoxically, reinforces the vulnerability of these women. In this context, journeys in search of help in legal and police contexts are permeated by intense feelings and suffering, in addition to progressive and cyclical expressions of violence^(16)^.

It is necessary to reflect on the complex interaction between socioeconomic norms and gender, since challenging structural elements of society that influence violence and shape gender roles can trigger other stressors. These, in turn, appear in the justifications presented by the interviewed women, with the risk of reducing the complexity inherent in the gender role expected in society. Furthermore, from the perspective of hegemonic masculinity, the social *status quo* would be maintained and, once again, women would be blamed^(8,9)^.

This understanding favors discussions about the importance of promoting protective and emancipatory aspects, such as the expansion of rights and economic security, autonomy, adequate educational level and effective health care, resuming the potential of nursing consultations in PHC to detect, advise and intervene in IPV situations^(12)^.

From the interpretation of the data revealing the phenomenon of *“Experiencing the request to revoke an emergency protective order*,” the professionals at the police station claimed that, unlike other rights violations they monitor, domestic violence against women is heavily emotionally influenced. This factor becomes a barrier to breaking off the relationship, whether through the acceptance of apologies and expressions of regret, or through social barriers related to marital status, financial difficulties, and responsibility for children^(17)^.

Similarly to this research, studies conducted in the United States on the revocation of Protective Orders found that, when formalizing petitions for these orders, women expressed fear and, subsequently, incorporated concepts of romantic love, reconciliation, and the potential for transformation. This process culminated in a rhetorical effort to rebuild the perpetrator’s image, valuing paternity and the partner’s promises of change - the cycle of violence^(18,19)^.

Therefore, the reasons for revoking the orders, as in this investigation, transcend the mere perception of economic dependence on intimate partners, since they involve multidimensional factors^(10)^. These factors justify the adverse feelings that permeate the revocation process, reported by the women involved in this phenomenon. This fact demonstrates that conscious choices to maintain the relationship are often based on the belief that facing the possible consequences of leaving the relationship could result in worsening the situation, as an adaptive behavior stemming from cognitive distortions^(20,21)^, configuring a cyclical journey experienced by these women and confirmed by professionals at the police station.

The no-contact condition imposed by the emergency protective order, according to the law, creates a feeling of harm to the perpetrator’s paternity. Therefore, legal proceedings involving children require greater attention to ensure the safety and well-being of both the child and the mother, who was once victimized^(17)^. The belief that the children’s needs are above their own guides these women to remain in the relationship, even if it means exposing them to the risk of revictimization^(17,18)^.

Although they have requested the revocation of the EPO, interpretation of the data reveals that they recognize the occurrence of different types of violence, including physical aggression, often characterized by brutal acts with lasting physical and emotional injuries, increasing the impacts on mental health, such as depression and anxiety^(22)^. The recognition of psychological and moral violence, in turn, configures the most painful and insidious type of violence, with repercussions that reverberate on self-esteem and mental health, resulting in psychological symptoms and post-traumatic stress disorder. It constitutes a subtle and suffocating system of control and power, marked by submission and fear, plunging these women into a spiral of anguish and vulnerability^(23)^.

Furthermore, the collected reports reveal a violent family dynamic, cyclical and progressive in nature, which impacts the woman’s ability to establish connections and hinders the ability to ask for help. Furthermore, this pattern reverberates in other relationships and social interactions within the family, weakening the parental role^(24)^. These findings refer to the assumptions of symbolic interactionism, which postulates that human beings act in the world according to the meanings attributed in social interactions, shaped by their communicative capacity^(14)^.

Additionally, alcohol and other psychoactive substances are strongly associated with domestic violence against women. The patterns of violence perpetrated by intoxicated men are unpredictable and illogical, triggering an erratic perception of guilt in victims, who are unable to foresee or prevent such acts^(25)^. In this context, living conditions and social relationships direct individuals’ perceptions of their lifestyles^(14)^.

Although the findings point to male jealousy as a trigger for conflict and violence, female jealousy can also play this role. However, there is a socially acceptable justification that male jealousy is a reactive action to women’s behavior, a culpable blaming^(26)^. This idea, stemming from hegemonic masculinity, reinforces the stereotypes and stigmas surrounding the issue of violence against women.

The factors previously discussed and linked to the personal history of the perpetrators, influenced by sociocultural and family perspectives, may be an explanatory variable for domestic violence in adulthood, with implications for future parenting practices^(27)^.

Emotions play a mediating role in meaning, especially when considering SI. Fear, guilt, and attachment are reinterpreted within the dynamics of the relationship, and remaining in a risky situation becomes meaningful for women experiencing violence, reinforcing their emotional attachment to the perpetrators. Understanding the emotional and psychological nuances of these women, combined with support strategies, is essential for nursing care that aims to break this perverse cycle^(14,23,24)^.

Health care resources must be combined with law enforcement to provide society with due care, since domestic violence constitutes a violation of human rights, in line with the global assumptions of eradicating violence against women^(1,2)^. In this sense, an approach that considers not only the legal aspects, but also the meanings and symbolic dynamics that support these attitudes is needed^(14)^.

The perception of risk posed by a violent relationship motivates women to seek help, which can be justified by the escalation of violence, the desire to protect their children, the rupture of intergenerational violence, the feeling of fear for their own life and/or the lives of their children, and changes in the perception of the romantic bond with the perpetrator, factors that converge with the findings of the present study^(28)^. This process highlights that meanings transform according to the interpretation that the person makes of the events of their daily life, always immersed in social relationships^(14)^.

The courage of women experiencing violence in confronting the legal system reflects their desire to end the spiral of violence by making the violation of their rights public. The specific classification of domestic violence in the legal codes of several countries reflects the need to hold perpetrators accountable, countering the culture of impunity and ensuring the protection of victims^(29)^. To ensure this protection, the Maria da Penha Law in Brazil provides for the establishment of emergency protective orders upon request by victims, triggering the activation of other judicial mechanisms when they appeal to the Police Station^(10)^.

The silence of many women and the revocation of protective orders reflect a pattern of survival that should not be interpreted as passivity. Instead, it should be understood as a response to conditions of high vulnerability, shaped by multiple factors, such as communication styles, emotional self-regulation strategies, conflict resolution skills, and perception of facts. The woman’s choice to remain in the abusive relationship can be seen as an attempt to cope with the uncertainty and associated risks, especially when institutional and social support is limited^(20)^. In light of the Symbolic Interactionism, this behavior is shaped by the meanings these women attribute to their experiences^(14)^.

Many women experience cognitive dissonance at the first signs of violence, oscillating between denying the danger and justifying the behavior^(20)^. This judgment is a mental process profoundly influenced by symbolic interactions^(14)^, such as messages received from their social context, especially from the perpetrator and their children.

Misconceptions and prejudices perpetuate stereotypical perceptions, subjugating women’s autonomy at critical moments when they could exercise it. Systematic and formal support has been shown to be twice as effective in leaving violent relationships in the following two years, compared to those who did not receive such services^(30)^. Furthermore, other types of violence had not been described, reinforcing the idea that this phenomenon is naturalized in society, especially in relation to traditional masculinities and femininities that reinforce stereotypical behaviors^(8,9)^.

A structural and cultural shift in how violence is perceived is needed, often masked by complex emotional dynamics. Investment should be made in preventive and supportive strategies that empower women to regain their autonomy and safety, fostering the role of primary care nurses in supporting women experiencing violence.

### Study Limitations

A limiting factor of the study is the specificity of the approach, which focused on women experiencing violence who searched the police station to request the revocation of their emergency protective order. Other aspects of the complex universe of women experiencing violence limit the generalization of the results, such as those who abandoned legal proceedings referred to the Court, those who did not formally revoke the protective order and reframed their experiences, as well as victims of femicide.

### Contributions to the Field of Nursing, Health or Public Policy

The study contributes with a comprehensive approach to the topic of domestic violence against women, highlighting the potential of healthcare professionals, especially PHC nurses, who, through their communication practices, can help these women reframe their lives. The study reinforces the gender rights perspective, promoting women’s autonomy in decision-making and protecting them from revictimization - a crucial role for nursing in the quest to eradicate this violence. The aim is to contribute to the field of care, from an interdisciplinary perspective, combined with updated law enforcement policies, for an approach free from stereotypical understandings that tend to subjugate autonomy.

## FINAL CONSIDERATIONS

The interpretation of the experiences of women experiencing domestic violence who requested the revocation of emergency protective orders reveals the complexity and magnitude of the challenges they face. When opting to have the orders revoked, they consider the repercussions of the decision not only for themselves but also for their children and the perpetrator. They are plagued by mixed feelings, such as guilt, regret, and sadness, even as they acknowledge the violent experience and the factors that contribute to its continuation.

Difficulties in understanding and supporting the criminal justice process, coupled with the persistence of the ideal of romantic love and the parental bond with the perpetrators, are frequent motivations for revoking protective orders. In many cases, women resist completely breaking away from the violent family dynamic, believing that maintaining it ensures some kind of emotional or material stability for their children or the family unit.

However, strategies for redefining their lives emerge significantly, revealing that, even when faced with an adverse scenario and complex decisions, these women demonstrate intention in rebuilding their trajectories. This process doesn’t occur linearly or immediately; it manifests in the search for autonomy, the strengthening of faith, and the construction of new perspectives on life, indicating movements of overcoming and resistance.

It is worth noting that the theoretical frameworks adopted - SI and GT - were useful for interpreting the experiences of women experiencing domestic violence, enabling the visibility of the symbolic and emotional dynamics influencing their decisions and perceptions. Further studies in this area can contribute to improving care, promoting sensitive and effective interventions that lead to breaking the cycle of violence and building new social possibilities for women.

## Data Availability

The dataset relating to the results processed in the NVIVO Plus *software*, version 11, is available at: https://doi.org/10.48331/SCIELODATA.RRXQOX.
