# Supplementary material for: Experiences of women facing intimate partner violence during the revocation of protective orders
Source: Rev Esc Enferm USP. 2025 Dec 5;59:e20250010. doi: 10.1590/1980-220X-REEUSP-2025-0010en (PMC12678001; doi:10.1590/1980-220X-REEUSP-2025-0010en)
Supplement: Translation

# Translation: Vivências de mulheres em situação de violência por parceiros íntimos na revogação da medida protetiva^*^

**DOI:** 10.1590/1980-220X-REEUSP-2025-0010pt

**Authors:** Paula Sales Rodrigues, Juliana Ribeiro da Silva Vernasque, Aline Pereira de Souza, Viviane Boacnin Yoneda Sponchiado, Fabiana Veronez Martelato Gimenez, Maria José Sanches Marin

**Affiliations:** 1 Faculdade de Medicina de Marília, Marília, SP, Brasil.; 2 Faculdade de Medicina de Botucatu, Programa de Pós-graduação de Doutorado em Enfermagem, Botucatu, SP, Brasil.; 3 Polícia Civil do Estado de São Paulo, Delegacia de Defesa da Mulher, Marília, SP, Brasil.

**Keywords:** Violência Doméstica, Violência contra a Mulher, Violência por Parceiro Íntimo, Direitos de Gênero, Teoria Fundamentada

## Abstract

**Objetivo:**

Interpretar as vivências de mulheres em situação de violência, que solicitam revogação da medida protetiva de urgência.

**Método:**

Utilizou-se a Teoria Fundamentada nos Dados (TFD), de Strauss e Corbin, e o Interacionismo Simbólico, na Delegacia da Mulher de um município paulista, por meio de entrevistas com 28 participantes de dois grupos amostrais: mulheres em situação de violência e profissionais da delegacia.

**Resultados:**

Os resultados foram organizados em categorias relacionadas aos componentes do modelo teórico, “*Vivenciando a solicitação da retirada da medida protetiva de urgência*”, evidenciando os desafios emocionais e contextuais enfrentados pelas mulheres na busca por ajuda formal. Esses desafios são permeados por sentimentos ambivalentes, experiências de revitimização e diferentes estratégias de enfrentamento para ressignificar suas trajetórias de vida.

**Considerações Finais:**

A revogação relaciona-se às consequências familiares - culpa, arrependimento e tristeza -, apesar de reconhecerem a violência. A incompreensão do sistema judicial e o vínculo afetivo com o perpetrador demonstram a necessidade de contemplar os significados simbólicos das interações na atuação da enfermagem da Atenção Primária.

## INTRODUÇÃO

As lideranças de diferentes países em todo o mundo delinearam estratégias significativas com vistas ao desenvolvimento sustentável, realizando ações que almejam fomentar a equidade em diversos contextos, em conformidade com os direitos e a dignidade humana^(1)^. Nessa perspectiva, pela primeira vez na história, uma agenda de desenvolvimento global investe na urgência da igualdade de gênero enfatizada por intermédio da Meta 5 dos Objetivos de Desenvolvimento Sustentável (ODS), com implicações em diversas dimensões, seja em educação, economia, saúde, práticas culturais nocivas, como os casamentos forçados e na erradicação de todas as formas de violência contra as mulheres e meninas^(2)^.

Notavelmente, o parceiro íntimo emerge como o principal perpetrador da violência^(3)^. Mundialmente, estima-se que entre 38% e 50% dos homicídios de mulheres ocorram pelas mãos de parceiros íntimos^(4)^, resultantes de um padrão contínuo de abuso, faltando uma compreensão mais significativa sobre os contextos e as motivações que influenciam na decisão de permanecer ou romper o relacionamento violento^(5)^.

No Brasil, país que ocupa o 5º lugar mundial em feminicídios, em média, uma mulher é morta a cada quatro horas, o que totaliza uma taxa diária de 4,3 homicídios femininos por 100 mil mulheres, quase o dobro da média global^(6)^. Essa realidade é mais pronunciada em contextos socioeconômicos desfavoráveis, frequentemente invisibilizados e estigmatizados. Portanto, os esforços para alterar as circunstâncias que essas mulheres enfrentam ainda são insuficientes^(7)^.

A violência perpetrada por parceiros íntimos (VPI) é um fenômeno que afeta tanto homens quanto mulheres. No entanto, este estudo concentra-se na violência de parceiros íntimos cometida por homens contra mulheres, especificamente aquelas que estão em união estável ou coabitantes, cisgêneros, heterossexuais e sexo biológico feminino. É importante ressaltar que, na América Latina e no Caribe (ALC), incluindo o Brasil, os discursos discriminatórios e posturas aparentemente protetoras, expressos por meio do sexismo hostil ou benevolente, reforçam estereótipos de gênero normativos que restringem a autonomia das mulheres^(8,9)^.

Frequentemente, após a formalização da denúncia que qualifica criminalmente o perpetrador, por meio da Lei Maria da Penha (2006), a medida protetiva de urgência é instaurada e o perpetrador é impedido de manter contato ou se aproximar dessa mulher. A medida só poderá ser revogada judicialmente, mesmo que haja qualquer solicitação de sua revogação^(10)^.

Destaca-se que a maioria das intervenções está voltada apenas para a segurança das mulheres em situação de violência doméstica, sem considerar a totalidade dos desdobramentos e das complexidades decorrentes do contato contínuo dessas famílias com os perpetradores. Tais complexidades decorrem das dinâmicas interacionais, da natureza progressiva e cíclica tanto das tentativas de rompimento quanto das relações parentais, elementos imprescindíveis para construção de intervenções efetivas que impactem a experiência das famílias que buscam por ajuda formal^(7,10,11)^.

Ademais, o fenômeno da VPI e a posterior revogação das medidas protetivas configuram uma problemática complexa^(10)^, que demanda uma abordagem interdisciplinar. Por isso, é fundamental que o setor da saúde amplie sua compreensão sobre o tema e fortaleça sua atuação na prevenção desse agravo, na intervenção e na promoção da saúde, sendo a atenção primária à saúde (APS), por meio das unidades de saúde, a referência de cuidado mais próxima e potencial para mulheres em situação de violência. As consultas de enfermagem podem desempenhar papel estratégico na resposta à VPI, ao favorecer a prevenção e a intervenção da VPI com base no cuidado longitudinal das famílias^(12)^.

O estudo busca contribuir para o avanço no campo do cuidado ao evidenciar as nuances frequentemente invisibilizadas nas análises tradicionais sobre a revogação de medidas protetivas por mulheres atendidas nos serviços especializados. Nessa perspectiva, esta investigação busca interpretar as vivências de mulheres em situação de violência, que solicitam revogação da medida protetiva de urgência, por meio da pergunta de pesquisa: quais as vivências de mulheres que solicitam a revogação da medida protetiva?

## MÉTODO

### Desenho de Estudo

Este estudo qualitativo utilizou como método a Teoria Fundamentada nos Dados (TFD) proposta por Strauss e Corbin^(13)^, e como referencial teórico, o Interacionismo Simbólico (IS)^(14)^. A TFD (que tem como base o IS) consiste em um método indutivo-dedutivo voltado para interpretação de vivências das pessoas inseridas em um determinado contexto social e preza pela criação de teorias fundamentadas sobre o fenômeno em estudo, que é expresso pelo modelo paradigmático. O IS considera a realidade social construída pela interação simbólica entre os indivíduos, em que se atribuem significados às coisas e aos eventos com base nas interações sociais, motivadores de suas ações e interpretações do mundo^(14)^.

A condução do estudo seguiu os pressupostos do *Consolidated Criteria For Reporting Qualitative Research* (COREQ)^(15)^.

### Local do Estudo

O local foi o Departamento de Polícia Judiciária de São Paulo Interior (DEINTER 4) em uma de suas seccionais, que contemplava uma Delegacia de Defesa da Mulher (DDM), localizada em um município de médio porte do Centro-Oeste do Estado de São Paulo, Brasil.

### Participantes e Critérios de Seleção

O estudo envolveu mulheres acima de 18 anos em situação de violência com registro de boletim de ocorrência que vieram à Delegacia para solicitar a revogação da MPU no período de setembro de 2021 a julho de 2022. A expressão de gênero não foi considerada critério de inclusão ou exclusão, assumindo-se, implicitamente, a expressão de gênero feminina. Foram excluídas aquelas que apresentaram qualquer comprometimento na capacidade de fornecer informações. O primeiro contato foi realizado pelos profissionais da delegacia que, ao prestarem atendimento, informavam sobre o estudo e, caso houvesse interesse e disponibilidade, a pesquisadora - doutoranda com vasta experiência profissional na área da saúde da mulher na atenção primária e na técnica de entrevistas qualitativas - era acionada. A pesquisadora aproximava-se da mulher, acolhia suas necessidades e explicitava as orientações sobre a participação na pesquisa. Foi utilizada a estratégia de permanência em plantões semanais às segundas e sextas-feiras para maior captação. As mulheres foram selecionadas progressivamente até se observar a ausência de novas informações para a compreensão do fenômeno em pauta, definido por meio de anuência dos autores.

De acordo com a amostragem teórica definida pela TFD^(13)^, desenvolveram-se conceitos que direcionaram os pesquisadores a novos questionamentos e à elaboração de hipóteses que foram respondidas na nova coleta de dados. Portanto, esse novo processo se deu concomitantemente à coleta e à análise dos dados, contando com o suporte dos memorandos. Percebeu-se que, por um lado, as mulheres se sentiam constrangidas de retornar à delegacia para o pedido de revogação, mas, , por outro lado, observou-se o grande envolvimento dos profissionais da delegacia, na tentativa de acolher essas mulheres, apontando para as consequências de voltar ao convívio com o perpetrador. Assim, estabeleceu-se o segundo grupo amostral composto por oito profissionais, ou seja, pela totalidade em atividades na Delegacia de Defesa da Mulher (DDM), sem recusas.

### Coleta E Organização Dos Dados

Para adequação do roteiro semiestruturado, foram realizadas três entrevistas piloto, em visitas domiciliares e na delegacia, resultando em pequenas adaptações. No primeiro grupo amostral, foram realizadas 20 entrevistas, que seguiram o roteiro semiestruturado com questões sobre as repercussões da medida protetiva, a decisão da sua retirada, os significados e sentimentos envolvidos. No decorrer das entrevistas, novos questionamentos foram incorporados, incluindo a solicitação para que as participantes descrevessem, detalhadamente, o evento que motivou o pedido da medida protetiva. A estratégia considerou que, no momento da retirada, as mulheres se expressaram de forma divergente ao conteúdo inicial do registro de ocorrência, influenciadas por múltiplos fatores emocionais, sociais e contextuais.

As entrevistas duraram, em média, 40 minutos, e foram acordadas por meio de contato telefônico para definir o local e horário de preferência das entrevistadas, considerando a necessidade de privacidade e segurança tanto da entrevistada quanto da pesquisadora. Assim, foram realizadas quatro entrevistas no domicílio, com a garantia de que os parceiros não estariam presentes, e as 16 restantes ocorreram em sala privada da delegacia.

Para o segundo grupo amostral, foram realizadas oito entrevistas agendadas, no local de trabalho, seguindo um roteiro semiestruturado contendo, além dos dados de identificação, questão sobre como eles percebem a situação das mulheres que solicitam a revogação da medida protetiva de urgência durante o atendimento na delegacia. Essas entrevistas tiveram duração média de 30 minutos, foram áudio gravadas com uso do MP3 *Player*, conduzidas e transcritas na íntegra pela pesquisadora principal, doutoranda com experiência profissional na área da saúde da mulher e na técnica de entrevistas qualitativas. A transcrição ocorreu logo após a realização de cada entrevista, dando início ao processo de análise para identificar a necessidade de aprofundamento nos dados, assim como proposto pela TFD.

### Análise Dos Dados

Em conformidade com as etapas do método da Teoria Fundamentada nos Dados (TFD) Straussiana^(13)^, a análise teve início com a codificação aberta. Nessa fase, os dados foram examinados minuciosamente, linha a linha, por meio de constantes questionamentos, com a finalidade de conceitualizar os significados expressos pelos participantes, permitindo compreender o que cada dado representa. Na segunda etapa, com a codificação axial, os dados foram reagrupados conforme suas relações e conexões, desenvolvendo explicações mais abrangentes sobre o fenômeno investigado. Este processo visa torna-los mais direcionados e conceituais, por intermédio de um movimento indutivo e dedutivo, que possibilita a identificação de padrões e relacionamentos entre os dados. Nesta etapa, houve uma integração entre as categorias, e o ponto principal foi a descoberta da categoria central ou fenômeno. Na codificação seletiva, foram escolhidas as categorias mais relevantes que integraram os dados em uma teoria mais abrangente^(13)^; o *software* NVIVO Plus, versão 11 foi utilizado.

Nesse contexto, tem-se o modelo paradigmático, abrangendo os seguintes componentes: condições, ações-interações e consequências. As condições referem-se às razões ou explicações pelas pessoas do porquê/como respondem às situações problemáticas. As ações/interações são significados atribuídos por elas aos eventos/situações problemáticas vivenciadas e como manejam/alcançam seu objetivo. As consequências são resultantes das ações/interações^(14)^.

Salienta-se que todo o processo de análise dos dados foi realizado, inicialmente, pela pesquisadora principal e, na sequência, analisado por uma das pesquisadoras com experiência na técnica de análise, ocasião em que foram realizados apontamentos e reflexões que levaram ao consenso sobre os códigos, categorias e subcategorias. A validação da interpretação dos dados ocorreu por meio do retorno às mulheres e aos profissionais da delegacia, bem como ao grupo de pesquisadores envolvidos, com a apresentação dos resultados e das reflexões acerca das inter-relações que culminaram no modelo teórico.

### Aspectos Éticos

O estudo integra o Projeto de Pesquisa - “A violência doméstica contra as mulheres: vivências e repercussões com a solicitação de revogação da medida protetiva de urgência” - aprovado pelo Comitê de Ética e Pesquisa (CEP) da Instituição proponente, conforme o Parecer nº 4.735.433. O Termo de Consentimento Livre e Esclarecido foi obtido de todos os participantes envolvidos no estudo, por escrito, pessoalmente. Soma-se, aos preceitos éticos, a substituição dos nomes dos entrevistados para menção das falas no texto, sendo M (Mulher) e D (profissional da delegacia), seguido do número referente à ordem de realização da entrevista.

## RESULTADOS

Na caracterização das 20 mulheres em situação de violência, identificou-se prevalência na faixa etária de 36 a 45 anos, autodeclaração da cor (cor parda ou negra), com conclusão do ensino médio. Quanto aos profissionais, a maioria é de mulheres na faixa etária acima de 40 anos, que se declararam de cor branca, com tempo médio de atuação na Delegacia de Defesa da Mulher (DDM), entre um e cinco anos. O processo de análise dos dados suscitou 1.192 códigos substantivos, 16 subcategorias e 4 categorias. No Quadro 1, observa-se a distribuição das categorias e respectivas subcategorias, que culminam no fenômeno: *“Vivenciando a solicitação da retirada da medida protetiva de urgência”*.

**Quadro 1:** Distribuição das Categorias e Subcategorias do Fenômeno “Vivenciando a solicitação da retirada da medida protetiva de urgência” – Marília, SP, Brasil, 2025.

Fenômeno: *“Vivenciando a solicitação da retirada da medida protetiva de urgência”*		
Componente do modelo	Categorias	Subcategorias
Condição	Contextualizando as consequências pela formalização da denúncia	• Pontuando consequências na vida dos filhos • Revelando consequências na vida do agressor • Sentindo consequências na própria vida • Sentindo culpa, arrependimento, medo e tristeza
Ação-interação	Reconhecendo a dinâmica cíclica da violência e seus condicionantes	• Sofrendo diferentes tipos de violência • Identificando comportamentos que levam à violência • Vivenciando a violência repetidamente • Relacionando ao uso abusivo de álcool e/ou outras drogas
Ação-interação	Justificando a revogação da medida protetiva de urgência	• Acreditando que a violência não vai mais ocorrer • Considerando que não foi agressão • Desconhecendo os pressupostos da Lei Maria da Penha • Vivenciando a impunidade • Sentindo amor, carinho e afeto
Consequência	Buscando caminhos para ressignificar a vida	• Buscando viver independentemente do agressor • Identificando elementos para efetiva rede de cuidados • Tendo fé, força e esperança

Elaboração própria, 2025.

Na interpretação do fenômeno central *“Vivenciando a solicitação da retirada da medida protetiva de urgência”*, considera-se como *condição* a categoria: “Contextualizando as consequências pela formalização da denúncia”; o componente *ações-interações* foi verificado nas categorias: “Reconhecendo a dinâmica cíclica da violência e seus condicionantes” e “Justificando a revogação da medida protetiva de urgência”. Foi definido como *consequência* a categoria: “Buscando caminhos para ressignificar a vida”.

Neste contexto, foi possível constatar que, após a realização do boletim de ocorrência e da solicitação de medida protetiva de urgência, as mulheres experienciam repercussões em suas próprias vidas, em seus filhos e na vida do perpetrador, provocando um misto de emoções, além de aflorarem dificuldades e necessidades que carecem de enfrentamentos, o que nem sempre é possível diante dos recursos emocionais e sociais dos quais dispõem.

Apesar da condição de solicitação da retirada da medida protetiva, elas reconhecem a existência da violência manifestada de diferentes formas, com destaque para a agressão física, a violência psicológica e moral, bem como a reiteração implacável desses episódios. Ademais, as motivações elencadas por essas mulheres para os eventos violentos referem-se à demanda por separação, interligações com o uso de substâncias psicoativas, como álcool e/ou drogas ilícitas. Porém, nesse movimento de ação e interação, buscam justificar a solicitação de revogação da medida protetiva de urgência, sugerindo uma oscilação entre o desejo de proteção e o pesar por suas decisões. Como consequência, constata-se a intencionalidade de superação dessa condição por meio da busca pela independência do perpetrador e o apego à religiosidade.

As interpretações do fenômeno também se sustentam nas observações dos profissionais da Polícia Judiciária, que destacam a gravidade progressiva dos episódios de violência e a utilização da medida protetiva como estratégia de proteção e, contraditoriamente, de poder e controle sobre o perpetrador.

A análise permitiu a elaboração do modelo paradigmático apresentado (Figura 1) a partir da Teoria Fundamentada nos Dados. Essa construção surge da interpretação do fenômeno central - *“Vivenciando a solicitação da retirada da medida protetiva de urgência” -* como um processo experiencial cíclico e dinâmico de tomada de decisão, influenciado e transversalizado por dimensões sociais, emocionais e contextuais.

**Figura 1:** Fenômeno “Vivenciando a solicitação da retirada da medida protetiva de urgência”. Marília, SP, Brasil, 2025.

### Condição - Contextualizando as Consequências Pela Formalização da Denúncia

A realização do boletim de ocorrência e da solicitação de medida protetiva de urgência traz consequências para a vida dessas mulheres, de seus filhos e, também, para os próprios perpetradores. Os filhos, mesmo querendo a proteção de suas mães, vivenciam a ausência do pai e sofrem com isso. Os perpetradores utilizam-se do desejo de regressar ao convívio familiar, expressados e relatados pelas mulheres pelos pedidos de reconciliação, temor da prisão e o impacto negativo no vínculo profissional. Para as mulheres, os registros de ocorrência suscitaram impactos emocionais descritos por sentimentos diversos, como culpa, arrependimento, medo, frustração e vergonha por ter realizado a denúncia e ter iniciado o processo judicial. Para os profissionais da delegacia, aumentam-se as atenções em virtude dos riscos de coação para a revogação das MPU.

*[...] a minha pequena essa semana teve febre, ela perguntou: mãe, quando o papai vai voltar.* (M4)

*[...] nós estamos bem mas muito chateado com o que aconteceu e com medo dele ser preso.* (M11)

*[...] ele ficou desesperado, mandava um monte de mensagem pedindo perdão para voltar para casa [...]* (M17)

*Do fato de eu ter que vir aqui fazer o boletim de ocorrência, eu me senti muito envergonhada perante as pessoas.* (M13)

*[...] quando elas voltam para retirar a medida, eu entendo que se arrependeram, muitas vezes, perdoaram. [...]eu acredito, que elas seriam coagidas a solicitar a retirada, mas geralmente, alegam a cabeça quente mesmo e o perdão.* (D3)

*[...] há preocupação em saber se de fato não está sendo coagida[...]* (D6)

### Ação-Interação --Reconhecendo a Dinâmica Cíclica da Violência e Seus Condicionantes

Apesar de solicitarem a revogação da medida protetiva, as entrevistadas reconhecem que passaram por diferentes tipos de violência. A violência física manifestou-se em atos, como socos, chutes, espancamentos e estrangulamentos, deixando perceptível a maldade e crueldade do ato. A violência patrimonial manifestou-se em formas distintas e caracterizadas pela destruição de pertences e patrimônio, acarretando prejuízos. As violências psicológica e moral foram demonstradas por falas do perpetrador carregadas de ameaças e chantagens emocionais, termos ofensivos, desvalorizantes e depreciativos, até mesmo com a aparência física.

Os relatos destacam a dinâmica da conjugalidade baseada na recorrência da violência e negligência a medidas judiciais anteriores. Para os profissionais da Polícia Judiciária, há a sensação de progressiva gravidade dos atos e aumento dos casos com padrões de repetição.

*Ele virava o meu braço assim, com tanta maldade, com tanta crueldade, que você via no olhar dele que queria quebrar mesmo.* (M6)

*Aí, continuou me chamando de vagabunda... você está gorda e feia [...]se eu largasse dele, ele me mataria.* (M1)

*[...]ele não fica agressivo comigo, mas ele joga objetos dentro de casa.* (M15)

*Porque tinha medida protetiva, mas ele nunca respeitou[...]* (M19)

*[...]porque o que era um xingamento, passou a ser um tapa, uma ameaça, quebrou o braço, quebrou um dente. A gente fala, o próximo vai ser um BO de seu óbito? Mas elas não se abalam.* (D2)

*[...]a violência doméstica é muito raro ela ir melhorando, o comum é que ela vai piorando.* (D6)

*Inclusive, ela acabou de falar que no início do ano, ela tinha feito uma protetiva e revogou porque ela acreditou nas promessas do marido.* (D7)

As mulheres destacam fatores que desencadeiam a violência, incluindo comportamentos, como ciúmes, possessividade, controle sobre a vítima, descontrole emocional, desconfiança, bem como solicitação da separação. Há relatos de vivência em situação de violência ao longo da vida, tanto por parte das mulheres quanto dos perpetradores, incluindo experiências de relacionamentos abusivos anteriores e traumas de infância. Associam, fortemente, o consumo abusivo de álcool e drogas ilícitas à violência, pelo perpetrador ou por ambos, resultando em perda de controle e comportamentos agressivos.

*[...]ele me ameaçou que se eu separasse ou pedisse o divórcio que faria da minha vida um inferno, que iria no meu trabalho, que tiraria o carro[...]* (M12)

*Ele é ciumento, muito conturbado [...] Idas e vindas, brigas, muito possessivo, ele enxergava coisas [...]* (M10)

*[...]quando ele bebe, ele tem lado machista, o gênio ruim, ele fala: eu sou ruim mesmo, não sou bom.* (M5)

*[...]tem um histórico violento [...]* M6

*Cada caso é um [...] muitos casos a vítima já vem de uma infância assim, para ela é normal.* (D8)

Nesse contexto, considera-se que a motivação para procurar a Delegacia se deu pela busca por alívio da situação.

*[...] aí criei coragem e vim na delegacia.* (M4)

*[...] para mim, a delegacia foi um ponto de alívio.* (M1)

*[...]você volta a ser útil! Um exemplo, uma instrução, de falar para a pessoa o que pode fazer para melhorar aquela situação [...]É uma experiência que eu vou levar para minha vida.* (D7)

### Ação-Interação - Justificando a Revogação da Medida Protetiva de Urgência

Apesar de reconhecerem a brutalidade da violência e que os fatores inerentes a ela, muitas vezes, compreendem hábitos que dificilmente serão eliminados, as mulheres entrevistadas justificam a revogação pela a crença de que a violência não se repetirá. Há uma busca em refazer a imagem do perpetrador, seja por admitir culpa, alegando precipitação e, até mesmo, alegando que mentiram no momento da denúncia. Outrossim, há o desejo de manutenção da família, arrependimento do perpetrador, sentimentos de amor, dependência emocional e financeira.

Os profissionais da Polícia Judiciária, por sua vez, indicam que a utilização da medida protetiva representa uma forma de exercer o poder e o controle sobre as ações do perpetrador. Ademais, o desconhecimento sobre a Lei Maria da Penha leva à utilização da MPU como um recurso de proteção temporária e de fácil revogação.

*E acontecer de novo não vai [...]ele foi bem claro que ele não queria fazer isso. Pediu desculpas [...]* (M2)

*Eu precipitei porque não precisava[...] estamos bem, indo na igreja, se um dia não der, eu separo e pronto.* (M12)

*[...] o vínculo emocional é muito forte.* (D4)

*Vou ter que falar a verdade, eu menti mesmo correndo o risco de ser processada [...]* (M11)

*[...]às vezes é a única forma que elas têm diante do que elas vivem, de ter um poder, o controle na mão, uma moeda de troca.* (D2)

*[...]ela se convence que a pessoa vai mudar porque ela quer se convencer, porque ela gosta da pessoa, então acha que fazendo a medida e depois tirar, vai resolver, e não, não resolve.* (D4)

### Consequências - Buscando Caminhos Para Ressignificar a Vida

As entrevistadas revelam o desejo de reconstruir suas vidas, com ou sem a presença do perpetrador, motivadas pela busca por independência financeira e alternativas legais, como o seguimento de processos civis de separação. Ainda há aquelas que mobilizaram recursos emocionais e espirituais para prosseguir com o parceiro, reconhecendo suas próprias potencialidades, a necessidade de diálogo e do afastamento de familiares “potencialmente prejudiciais”.

Os profissionais da Polícia Judiciária indicam a necessidade de uma rede de cuidados efetiva em casos de violência doméstica, visando à redução das revitimizações e à revogação de medidas protetivas.

*Quero trabalhar, entregar currículo. [...]estou com advogado e vou me separar.* (M1)

*[...] quero voltar a trabalhar, estando com ele ou não.* (M4)

*Comecei a fazer os cursos que eu queria fazer, a viver a minha vida [...] a pensar diferente, ver que eu posso fazer coisas sem ele.* (M3)

*[...] estamos conversando bastante, indo na igreja e respeitando um ao outro.* (M12)

*[...]eu acredito que Deus vai mudar o jeito dele [...]é o que tem me dado força, minha fé.* (M17)

*[...] eles precisam de tratamento psicológico mesmo [...]* (D2)

*[...] precisa ter uma equipe multidisciplinar para fazer o primeiro acolhimento[...]os profissionais de saúde, assistência social, e a defensoria pública, seria o necessário.* (D4)

## DISCUSSÃO

Entre as mulheres em situação de violência doméstica deste estudo, os dados socioeconômicos, como cor, faixa etária e escolaridade, revelam características de pleno desenvolvimento reprodutivo e potencial para ascensão econômica e social, o que, paradoxalmente, reforça as condições de vulnerabilidade dessas mulheres. Nesse contexto, os trajetos em busca de ajuda nos espaços jurídico-policiais são permeados por intensos sentimentos e sofrimentos, além de expressões progressivas e cíclicas da violência^(16)^.

Faz-se necessária uma reflexão sobre a interação complexa entre normas socioeconômicas e gênero, uma vez que desafiar elementos estruturantes da sociedade que incidem sobre a violência e moldam os papeis de gênero pode desencadear outros fatores estressores. Esses, por sua vez, aparecem nas justificativas apresentadas pelas mulheres entrevistadas, com riscos de redução de toda complexidade inerente ao papel de gênero esperado na sociedade. Além disso, na ótica da masculinidade hegemônica, haveria a manutenção do *status quo* social e, mais uma vez, a culpabilização da mulher^(8,9)^.

Essa compreensão favorece discussões sobre a importância da promoção de aspectos protetores e emancipadores, como a expansão dos direitos e segurança econômica, autonomia, adequado nível educacional e uma atenção à saúde efetiva, retomando a potencialidade das consultas de enfermagem na APS para detectar, aconselhar e intervir nas situações de VPI^(12)^.

Na interpretação dos dados das entrevistas que revelam o fenômeno *“Vivenciando a solicitação da retirada da medida protetiva de urgência”*, os profissionais da delegacia alegaram que, diferentemente de outras violações de direitos que acompanhavam, a violência doméstica contra as mulheres sofre grande influência emocional. Esse fator torna-se uma barreira para o rompimento da relação, seja pela aceitação dos pedidos de desculpas e demonstrações de arrependimento, seja por barreiras sociais relacionadas à conjugalidade, dificuldades financeiras e a responsabilidade sobre os filhos^(17)^.

Na mesma linha da presente investigação, estudos realizados nos Estados Unidos sobre a revogação de Ordens de Proteção identificaram que, ao formalizarem as petições dessas ordens, as mulheres expressavam temor e, posteriormente, incorporavam conceitos do amor romântico à reconciliação e ao potencial de transformação. Esse processo culminava em esforço retórico para reconstruir a imagem do perpetrador, valorizando a paternidade e as promessas de mudança do parceiro – o ciclo da violência^(18,19)^.

Portanto, as razões para a revogação das medidas, assim como nesta investigação, transcendem a mera percepção de dependência econômica dos parceiros íntimos, visto que envolvem fatores multidimensionais^(10)^. Esses fatores justificam a contrariedade de sentimentos que permeiam o processo de revogação, relatados pelas mulheres envolvidas nesse fenômeno. Tal fato demonstra que as escolhas conscientes de manutenção do relacionamento estão, frequentemente, na crença de que enfrentar as possíveis consequências de uma saída da relação poderia resultar na piora da situação, como um comportamento adaptativo advindo de distorções cognitivas^(20,21)^, configurando jornada cíclica vivenciada por essas mulheres e confirmada pelos profissionais da delegacia.

A condição de ausência de contato imposta pela medida protetiva de urgência, nos termos da lei, gera a sensação de prejuízos à paternidade do perpetrador. Por isso, os processos judiciais envolvendo crianças exigem maior atenção para garantir a segurança e o bem-estar da criança e da mãe, outrora vitimizada^(17)^. A crença de que a necessidade dos filhos estaria acima das suas próprias orienta a permanência dessas mulheres no relacionamento, mesmo que isso signifique exposição ao risco de revitimização^(17,18)^.

Embora tenham solicitado a revogação da MPU, a interpretação dos dados revela que elas reconhecem a ocorrência de diferentes tipos de violência, incluindo a agressão física, frequentemente caracterizada por atos brutais com lesões físicas e emocionais duradouras, aumentando os impactos sobre a saúde mental, como depressão e ansiedade^(22)^. O reconhecimento da violência psicológica e moral, por sua vez, apresenta-se como a tipificação de violência mais dolorosa e insidiosa com repercussões que reverberam na autoestima e saúde mental, resultando em sintomas psíquicos e transtorno do estresse pós-traumático. Configura-se como um sistema sutil e asfixiante de controle e poder, marcado pela submissão e medo, mergulhando essas mulheres em um espiral de angústia e vulnerabilidade^(23)^.

Outrossim, nos relatos coletados, observa-se uma dinâmica familiar violenta, de caráter cíclico e progressivo, que impacta na capacidade da mulher de estabelecer conexões e dificulta a solicitação de ajuda. Além disso, esse padrão reverbera em outras relações e interações sociais da família, fragilizando o papel parental^(24)^. Esses achados remetem aos pressupostos do interacionismo simbólico, o qual postula que os seres humanos agem no mundo segundo os significados atribuídos nas interações sociais, moldados por sua capacidade comunicativa^(14)^.

Adicionalmente, o álcool e demais substâncias psicoativas estão fortemente associados à violência doméstica contra as mulheres. Os padrões de violência perpetrada por homens embriagados são imprevisíveis e ilógicos, desencadeando uma errática percepção de culpa nas vítimas, que não conseguem prever ou evitar tais atos^(25)^. Nesse contexto, as condições de vida e as relações sociais direcionam a percepção dos indivíduos no seu modo de viver^(14)^.

Embora os resultados apontem o ciúme masculino como fator desencadeante de conflitos e violência, o ciúme feminino também pode desempenhar esse papel. Contudo, há uma justificativa socialmente aceitável de que o ciúmes masculino seria uma ação reativa ao comportamento da mulher, uma responsabilização culposa^(26)^. Essa ideia advinda da masculinidade hegemônica reforça os estereótipos e estigmas que envolvem a temática da violência contra as mulheres.

Os fatores anteriormente discutidos e atrelados ao histórico pessoal dos perpetradores, influenciado por perspectivas socioculturais e familiares, pode ser uma variável explicativa para a violência doméstica na idade adulta, com implicações para futuras práticas parentais^(27)^.

As emoções desempenham um papel mediador de significados, especialmente quando se considera o IS. O medo, a culpa e o apego são ressignificados dentro da dinâmica da relação, e a permanência em uma condição de risco passa a fazer sentido para mulheres em situação de violência, reforçando o apego emocional aos perpetradores. A percepção das nuances emocionais e psicológicas dessas mulheres, aliada a estratégias de apoio, é fundamental para uma assistência de enfermagem que visa promover o rompimento deste ciclo perverso^(14,23,24)^.

Os recursos da assistência à saúde devem ser aliados à aplicação da lei, a fim de proporcionar à sociedade a atenção devida, uma vez que a violência doméstica configura uma violação dos direitos humanos, alinhando-se aos pressupostos globais de erradicação da violência contra as mulheres^(1,2)^. Nesse sentido, é necessária uma abordagem que considere não apenas os aspectos legais, mas também os significados e as dinâmicas simbólicas que sustentam essas atitudes^(14)^.

A percepção do risco imposto por um relacionamento violento motiva as mulheres a buscar ajuda, o que pode ser justificado pela escalada da violência, pelo desejo de proteção dos filhos, pelo rompimento da intergeracionalidade da violência, pela sensação de medo em relação à própria vida e/ou à vida dos filhos, e por mudanças na percepção do vínculo romântico com o perpetrador, fatores que convergem com os achados do presente estudo^(28)^. Esse processo ressalta que os significados se transformam conforme a interpretação que a pessoa faz dos eventos de seu cotidiano, sempre imersos nas relações sociais^(14)^.

A coragem da mulher em situação de violência, ao enfrentar o sistema legal, reflete o desejo de pôr fim à espiral de violência, tornando pública a violação de seus direitos. A tipificação específica da violência doméstica nos códigos legais de diversos países reflete a necessidade de responsabilizar os perpetradores, contrapondo-se à cultura de impunidade e garantindo a proteção das vítimas^(29)^. Para garantir essa proteção, a Lei Maria da Penha, no Brasil, prevê a instauração de medidas protetivas de urgência, por meio da solicitação das vítimas, deflagrando a ativação dos demais mecanismos judiciais ao recorrer à Delegacia de Polícia Judiciária^(10)^.

O silêncio de muitas mulheres e a revogação das medidas protetivas refletem um padrão de sobrevivência que não deve ser interpretado como passividade. Em vez disso, deve ser compreendido como uma resposta às condições de alta vulnerabilidade, moldada por múltiplos fatores, como estilos de comunicação, estratégias de autorregulação emocional, habilidades de resolução de conflitos e percepção dos fatos. A escolha da mulher em manter o relacionamento abusivo pode ser vista como uma tentativa de lidar com a incerteza e os riscos associados, especialmente quando o suporte institucional e social é limitado^(20)^. À luz do Interacionismo Simbólico, esse comportamento é moldado pelos significados que essas mulheres atribuem às suas experiências^(14)^.

Muitas mulheres vivenciam uma dissonância cognitiva diante dos primeiros sinais de violência, oscilando entre negar o perigo e justificar o comportamento^(20)^. Esse julgamento é um processo mental profundamente influenciado pelas interações simbólicas^(14)^, como as mensagens recebidas do seu contexto social, sobretudo do perpetrador e dos filhos.

Equívocos e preconceitos perpetuam percepções estereotipadas, subjugando a autonomia das mulheres em momentos críticos em que poderiam exercê-la. O apoio sistemático e formal demonstrou ser duas vezes mais eficaz para a saída dos relacionamentos violentos nos dois anos subsequentes, em comparação com aquelas que não receberam esses serviços^(30)^. Além disso, outras tipologias de violência não foram descritas, reforçando a ideia de naturalização desse fenômeno na sociedade, especialmente em relação às masculinidades e feminilidades tradicionais que reforçam os comportamentos estereotipados^(8,9)^.

É necessário uma mudança estrutural e cultural sobre como a violência é percebida, muitas vezes ocultada por dinâmicas emocionais complexas. Deve-se investir em estratégias preventivas e de apoio que capacitem as mulheres a recuperar sua autonomia e segurança, favorecendo o protagonismo da Enfermagem da Atenção Primária no acompanhamento de mulheres em situação de violência.

### Limitações do Estudo

Considera-se um fator limitante do estudo a especificidade da abordagem, que se concentrou nas mulheres em situação de violência que recorreram à delegacia para solicitar a revogação da medida protetiva de urgência. Outros aspectos do complexo universo das mulheres em situação de violência limitam a generalização dos resultados, como aquelas que abandonaram os processos judiciais encaminhados ao Fórum, as que não revogaram formalmente a medida protetiva e ressignificaram suas vivências, bem como as vítimas de feminicídio.

### Contribuições Para a Área da Enfermagem, Saúde Ou Políticas Públicas

O estudo contribui com uma abordagem abrangente sobre a temática da violência doméstica contra as mulheres, destacando a potencialidade dos profissionais da saúde, em especial da enfermagem na APS, que nas suas práticas comunicativas podem auxiliar essas mulheres a ressignificar suas vidas. Reforça-se a perspectiva dos direitos de gênero, promovendo a autonomia das mulheres nas tomadas de decisões e na proteção contra a revitimização, um papel essencial para a enfermagem na busca pela erradicação dessa violência. Almeja-se contribuir com o campo do processo de cuidar, na perspectiva da interdisciplinaridade, aliado às políticas atualizadas de aplicação da lei para uma abordagem livre de compreensões estereotipadas que tendem a subjugar a autonomia.

## CONSIDERAÇÕES FINAIS

Na interpretação das vivências de mulheres em situação de violência doméstica que solicitaram a revogação das medidas protetivas de urgência, evidencia-se a complexidade e a magnitude dos desafios enfrentados por elas. Ao optarem pela retirada das medidas, elas avaliam as repercussões da decisão não apenas sobre si mesmas, mas sobre os filhos e o perpetrador, sendo atravessadas por sentimentos ambíguos, como culpa, arrependimento e tristeza, mesmo quando reconhecem a vivência violenta e os fatores que contribuem para sua continuidade.

As dificuldades em compreender e sustentar o processo judicial criminal, associadas à persistência do ideal do amor romântico e ao vínculo parental com os perpetradores, constituem motivações frequentes para a revogação das medidas protetivas. Em muitos casos, as mulheres resistem em romper completamente com a dinâmica familiar violenta, acreditando que sua manutenção assegura algum tipo de estabilidade emocional ou material aos filhos ou para o núcleo familiar.

Contudo, estratégias para ressignificar suas vidas emergem de forma significativa, revelando que, mesmo diante de um cenário adverso e de decisões complexas, essas mulheres demonstram intencionalidade na reconstrução de suas trajetórias. Esse processo não ocorre de maneira linear ou imediata, manifesta-se na busca por autonomia, no fortalecimento da fé e na construção de novas perspectivas de vida, indicando movimentos de superação e resistência.

Salienta-se que os referenciais teóricos adotados - IS e TFD - foram oportunos para a interpretação das vivências das mulheres em situação de violência doméstica, permitindo dar visibilidade às dinâmicas simbólicas e emocionais que influenciam suas decisões e percepções. Espera-se que novos estudos na área possam contribuir para a qualificação do cuidado, promovendo intervenções sensíveis e eficazes, que viabilizem a ruptura do ciclo de violência e a construção de novas possibilidades sociais para as mulheres.

## Data Availability

O conjunto de dados referentes aos resultados trabalhados no *software* NVIVO Plus, versão 11, encontram-se disponíveis em: https://doi.org/10.48331/SCIELODATA.RRXQOX.

# Translation: Experiencias de mujeres en situación de violencia por parte de sus parejas íntimas en la revocación de la medida de protección*

**DOI:** 10.1590/1980-220X-REEUSP-2025-0010es

**Authors:** Paula Sales Rodrigues, Juliana Ribeiro da Silva Vernasque, Aline Pereira de Souza, Viviane Boacnin Yoneda Sponchiado, Fabiana Veronez Martelato Gimenez, Maria José Sanches Marin

**Affiliations:** 1 Faculdade de Medicina de Marília, Marília, SP, Brasil.; 2 Faculdade de Medicina de Botucatu, Programa de Pós-graduação de Doutorado em Enfermagem, Botucatu, SP, Brasil.; 3 Polícia Civil do Estado de São Paulo, Delegacia de Defesa da Mulher, Marília, SP, Brasil.

**Keywords:** Violencia Doméstica, Violencia contra la Mujer, Violencia de Pareja, Derechos de Género, Teoría Fundamentada

## Abstract

**Objetivo:**

Interpretar las experiencias de mujeres en situación de violencia que solicitan la revocación de medidas de protección de urgencia.

**Método:**

Se utilizaron la Teoría Fundamentada de Strauss y Corbin (TF) y el Interaccionismo Simbólico en la Comisaría de la Mujer de un municipio del Estado de São Paulo, a través de entrevistas a 28 participantes de dos grupos muestrales: mujeres en situación de violencia y profesionales de la comisaría.

**Resultados:**

Los resultados se organizaron en categorías relacionadas con los componentes del modelo teórico «Experimentar la solicitud de revocación de una medida de protección de urgencia», poniendo de relieve los desafíos emocionales y contextuales a los que se enfrentan las mujeres que buscan ayuda formal. Estos desafíos están atravesados por sentimientos ambivalentes, experiencias de revictimización y diferentes estrategias de afrontamiento para resignificar sus trayectorias vitales.

**Consideraciones finales:**

La revocación se relaciona con consecuencias familiares - culpa, arrepentimiento y tristeza -, a pesar del reconocimiento de la violencia. La falta de comprensión del sistema judicial y el vínculo afectivo con el agresor evidencian la necesidad de considerar los significados simbólicos de las interacciones en la práctica de la enfermería de atención primaria.

## INTRODUCCIÓN

Los líderes de diferentes países de todo el mundo han diseñado estrategias significativas con miras al desarrollo sostenible, llevando a cabo acciones que buscan fomentar la equidad en diversos contextos, de conformidad con los derechos y la dignidad humanos^(1)^. En esta perspectiva, por primera vez en la historia, una agenda de desarrollo global invierte en la urgencia de la igualdad de género enfatizada a través de la Meta 5 de los Objetivos de Desarrollo Sostenible (ODS), con implicaciones en diversas dimensiones, ya sea en la educación, la economía, la salud, las prácticas culturales nocivas, como los matrimonios forzados, y en la erradicación de todas las formas de violencia contra las mujeres y las niñas^(2)^.

Cabe destacar que la pareja íntima emerge como el principal perpetrador de la violencia^(3)^. A nivel mundial, se estima que entre el 38 % y el 50 % de los homicidios de mujeres son cometidos por sus parejas íntimas^(4)^, como resultado de un patrón continuo de abuso, sin que se comprenda de manera más significativa los contextos y las motivaciones que influyen en la decisión de permanecer o romper la relación violenta^(5)^.

En Brasil, país que ocupa el quinto lugar mundial en feminicidios, en promedio, una mujer es asesinada cada cuatro horas, lo que suma una tasa diaria de 4,3 homicidios de mujeres por cada 100 mil mujeres, casi el doble del promedio mundial^(6)^. Esta realidad es más pronunciada en contextos socioeconómicos desfavorables, a menudo invisibilizados y estigmatizados. Por lo tanto, los esfuerzos para cambiar las circunstancias a las que se enfrentan estas mujeres siguen siendo insuficientes^(7)^.

La violencia perpetrada por parejas íntimas (VPI) es un fenómeno que afecta tanto a hombres como a mujeres. Sin embargo, este estudio se centra en la violencia de pareja íntima cometida por hombres contra mujeres, específicamente aquellas que están en una unión estable o conviven, son cisgénero, heterosexuales y de sexo biológico femenino. Es importante destacar que, en América Latina y el Caribe (ALC), incluido Brasil, los discursos discriminatorios y las posturas aparentemente protectoras, expresadas a través del sexismo hostil o benévolo, refuerzan los estereotipos de género normativos que restringen la autonomía de las mujeres^(8,9)^.

A menudo, tras la formalización de la denuncia que califica penalmente al agresor, mediante la Ley María da Penha (2006), se establece la medida cautelar de urgencia (MCU) y se impide al agresor mantener contacto o acercarse a la mujer. La medida solo puede ser revocada judicialmente, incluso si se solicita su revocación^(10)^.

Cabe destacar que la mayoría de las intervenciones se centran únicamente en la seguridad de las mujeres en situaciones de violencia doméstica, sin tener en cuenta la totalidad de las ramificaciones y complejidades derivadas del contacto continuo de estas familias con los agresores. Dichas complejidades se derivan de las dinámicas interactivas, de la naturaleza progresiva y cíclica tanto de los intentos de ruptura como de las relaciones parentales, elementos imprescindibles para la construcción de intervenciones efectivas que impacten en la experiencia de las familias que buscan ayuda formal^(7,10,11)^.

Además, el fenómeno de la violencia de pareja y la posterior revocación de las medidas cautelares constituyen un problema complejo^(10)^, que exige un enfoque interdisciplinario. Por ello, es fundamental que el sector de la salud amplíe su comprensión sobre el tema y fortalezca su actuación en la prevención de este agravio, en la intervención y en la promoción de la salud, siendo la atención primaria de salud (APS), a través de las unidades de salud, la referencia de atención más cercana y potencial para las mujeres en situación de violencia. Las consultas de enfermería pueden desempeñar un papel estratégico en la respuesta a la violencia de género, al favorecer la prevención y la intervención de la violencia de género basadas en la atención longitudinal de las familias^(12)^.

El estudio busca contribuir al avance en el campo de la atención al evidenciar los matices que a menudo pasan desapercibidos en los análisis tradicionales sobre la revocación de medidas cautelares por parte de las mujeres atendidas en los servicios especializados. Desde esta perspectiva, esta investigación busca interpretar las experiencias de las mujeres en situación de violencia que solicitan la revocación de la medida cautelar de urgencia, a través de la pregunta de investigación: ¿cuáles son las experiencias de las mujeres que solicitan la revocación de la medida cautelar?

## MÉTODO

### Diseño Del Estudio

Este estudio cualitativo utilizó como método la Teoría Fundamentada en los Datos (TFD) propuesta por Strauss y Corbin^(13)^, y como referencia teórica, el Interaccionismo Simbólico (IS)^(14)^. La TFD (que se basa en el IS) consiste en un método inductivo-deductivo orientado a la interpretación de las experiencias de las personas insertadas en un determinado contexto social y valora la creación de teorías fundamentadas sobre el fenómeno en estudio, que se expresa mediante el modelo paradigmático. El IS considera la realidad social construida por la interacción simbólica entre los individuos, en la que se atribuyen significados a las cosas y a los acontecimientos basándose en las interacciones sociales, motivadores de sus acciones e interpretaciones del mundo^(14)^.

La realización del estudio siguió los supuestos de los Criterios Consolidados para la Presentación de Informes de Investigación Cualitativa (COREQ)^(15)^.

### Lugar Del Estudio

El lugar fue el Departamento de Policía Judicial del Interior de São Paulo (DEINTER 4) en una de sus secciones, que incluía una Comisaría de Defensa de la Mujer (Delegacia de Defesa da Mulher - DDM), ubicada en un municipio de tamaño medio del centro-oeste del estado de São Paulo, Brasil.

### Participantes y Criterios de Selección

El estudio incluyó a mujeres mayores de 18 años en situación de violencia con denuncia policial registrada que acudieron a la comisaría para solicitar la revocación de la MCU en el periodo comprendido entre septiembre de 2021 y julio de 2022. La expresión de género no se consideró un criterio de inclusión o exclusión, asumiéndose implícitamente la expresión de género femenino. Se excluyó a aquellas que presentaban alguna limitación en su capacidad para proporcionar información. El primer contacto lo realizaron los profesionales de la comisaría que, al atender a las mujeres, les informaban sobre el estudio y, en caso de que hubiera interés y disponibilidad, se contactaba a la investigadora, una doctoranda con amplia experiencia profesional en el área de la salud de la mujer en la atención primaria y en la técnica de entrevistas cualitativas. La investigadora se acercaba a la mujer, atendía sus necesidades y le explicaba las orientaciones sobre la participación en la investigación. Se utilizó la estrategia de permanecer en turnos semanales los lunes y viernes para una mayor captación. Las mujeres fueron seleccionadas progresivamente hasta que se observó la ausencia de nueva información para la comprensión del fenómeno en cuestión, definido mediante el consentimiento de los autores.

De acuerdo con el muestreo teórico definido por la TFD^(13)^, se desarrollaron conceptos que orientaron a los investigadores hacia nuevas preguntas y la elaboración de hipótesis que fueron respondidas en la nueva recopilación de datos. Por lo tanto, este nuevo proceso se llevó a cabo simultáneamente con la recopilación y el análisis de los datos, con el apoyo de los memorandos. Se observó que, por un lado, las mujeres se sentían cohibidas de volver a la comisaría para solicitar la revocación, pero, por otro lado, se observó una gran implicación de los profesionales de la comisaría, en un intento de acoger a estas mujeres, señalando las consecuencias de volver a convivir con el agresor. Así, se estableció el segundo grupo muestral compuesto por ocho profesionales, es decir, la totalidad de los que trabajaban en la Comisaría de Defensa de la Mujer (DDM), sin rechazos.

### Recopilación y Organización de Datos

Para adaptar el guion semiestructurado, se realizaron tres entrevistas piloto, en visitas domiciliarias y en la delegación, lo que dio lugar a pequeñas adaptaciones. En el primer grupo muestral, se realizaron 20 entrevistas, que siguieron el guion semiestructurado con preguntas sobre las repercusiones de la medida de protección, la decisión de su retirada, los significados y los sentimientos involucrados. Durante las entrevistas, se incorporaron nuevas preguntas, incluida la solicitud de que las participantes describieran en detalle el evento que motivó la solicitud de la medida de protección. La estrategia consideró que, en el momento de la retirada, las mujeres se expresaron de manera divergente al contenido inicial del registro de la denuncia, influenciadas por múltiples factores emocionales, sociales y contextuales.

Las entrevistas duraron, en promedio, 40 minutos, y se acordaron por teléfono para definir el lugar y la hora preferidos por las entrevistadas, teniendo en cuenta la necesidad de privacidad y seguridad tanto de la entrevistada como de la investigadora. Así, se realizaron cuatro entrevistas en el domicilio, con la garantía de que las parejas no estarían presentes, y las 16 restantes se llevaron a cabo en una sala privada de la comisaría.

Para el segundo grupo de la muestra, se realizaron ocho entrevistas programadas en el lugar de trabajo, siguiendo un guion semiestructurado que contenía, además de los datos de identificación, preguntas sobre cómo perciben la situación de las mujeres que solicitan la revocación de la medida cautelar de urgencia durante la atención en la comisaría. Estas entrevistas tuvieron una duración media de 30 minutos, se grabaron en audio con un reproductor MP3, fueron realizadas y transcritas íntegramente por la investigadora principal, doctoranda con experiencia profesional en el área de la salud de la mujer y en la técnica de entrevistas cualitativas. La transcripción se realizó inmediatamente después de cada entrevista, dando inicio al proceso de análisis para identificar la necesidad de profundizar en los datos, tal y como propone la TFD.

### Análisis de Los Datos

De acuerdo con las etapas del método de la Teoría Fundamentada en los Datos (TFD) de Strauss^(13)^, el análisis comenzó con la codificación abierta. En esta fase, los datos se examinaron minuciosamente, línea por línea, mediante preguntas constantes, con el fin de conceptualizar los significados expresados por los participantes, lo que permitió comprender lo que representa cada dato.

En la segunda etapa, con la codificación axial, los datos se reagruparon según sus relaciones y conexiones, desarrollando explicaciones más amplias sobre el fenómeno investigado. Este proceso tiene como objetivo hacerlos más específicos y conceptuales, mediante un movimiento inductivo y deductivo, que permite identificar patrones y relaciones entre los datos. En esta etapa, se produjo una integración entre las categorías, y el punto principal fue el descubrimiento de la categoría central o fenómeno. En la codificación selectiva, se eligieron las categorías más relevantes que integraron los datos en una teoría más amplia^(13)^; se utilizó el software NVIVO Plus, versión 11.

En este contexto, se tiene el modelo paradigmático, que abarca los siguientes componentes: condiciones, acciones-interacciones y consecuencias. Las condiciones se refieren a las razones o explicaciones de las personas sobre por qué/cómo responden a las situaciones problemáticas. Las acciones/interacciones son significados que ellas atribuyen a los eventos/situaciones problemáticas vividas y cómo manejan/alcanzan su objetivo. Las consecuencias son el resultado de las acciones/interacciones^(14)^.

Cabe destacar que todo el proceso de análisis de los datos fue realizado, inicialmente, por la investigadora principal y, a continuación, analizado por una de las investigadoras con experiencia en la técnica de análisis, momento en el que se realizaron anotaciones y reflexiones que llevaron al consenso sobre los códigos, categorías y subcategorías. La validación de la interpretación de los datos se realizó mediante la retroalimentación a las mujeres y a los profesionales de la comisaría, así como al grupo de investigadores involucrados, con la presentación de los resultados y las reflexiones sobre las interrelaciones que culminaron en el modelo teórico.

### Aspectos Éticos

El estudio forma parte del Proyecto de Investigación “La violencia doméstica contra las mujeres: experiencias y repercusiones de la solicitud de revocación de la medida cautelar de urgencia”, aprobado por el Comité de Ética e Investigación (CEP) de la institución proponente, de conformidad con el Dictamen n.º 4.735.433. Se obtuvo el Consentimiento Libre y Esclarecido de todos los participantes involucrados en el estudio, por escrito y en persona. A los preceptos éticos se suma la sustitución de los nombres de los entrevistados para mencionar sus declaraciones en el texto, siendo M (Mujer) y D (profesional de la comisaría), seguido del número correspondiente al orden de realización de la entrevista.

## RESULTADOS

En la caracterización de las 20 mujeres en situación de violencia, se identificó una prevalencia en el rango de edad de 36 a 45 años, autodeclaración del color (pardo o negro) y finalización de la educación secundaria. En cuanto a los profesionales, la mayoría son mujeres de más de 40 años, que se declararon de raza blanca, con un tiempo medio de actuación en la Delegatura de Defensa de la Mujer (DDM) de entre uno y cinco años. El proceso de análisis de los datos dio lugar a 1192 códigos sustantivos, 16 subcategorías y 4 categorías. En el Cuadro 1 se observa la distribución de las categorías y sus respectivas subcategorías, que culminan en el fenómeno: “Experimentando la solicitud de retirada de la medida cautelar de urgencia”.

**Cuadro 1:** Distribución de las categorías y subcategorías del fenómeno “Experimentando la solicitud de retirada de la medida cautelar de urgencia” – Marília, SP, Brasil, 2025.

Fenómeno: *“Experimentando la solicitud de retirada de la medida cautelar de urgencia”*		
Componente del modelo	Categorías	Subcategorías
Condición	Contextualizando las consecuencias de la formalización de la denuncia	• Señalando las consecuencias en la vida de los hijos • Revelando las consecuencias en la vida del agresor • Sintiendo las consecuencias en la propia vida • Sintiendo culpa, arrepentimiento, miedo y tristeza
Acción-interacción	Reconociendo la dinámica cíclica de la violencia y sus condicionantes	• Sufriendo diferentes tipos de violencia • Identificando comportamientos que conducen a la violencia • Experimentando la violencia de forma repetida • Relacionándolo con el abuso de alcohol y/u otras drogas
Acción-interacción	Justificando la revocación de la medida cautelar de urgencia	• Creer que la violencia no volverá a ocurrir • Considerar que no fue una agresión • Desconocer los supuestos de la Ley María da Penha • Experimentar la impunidad • Sentir amor, cariño y afecto
Consecuencia	Buscando caminos para darle un nuevo sentido a la vida	• Buscar vivir independientemente del agresor • Identificar elementos para una red de cuidados eficaz • Tener fe, fuerza y esperanza

Elaborado por los autores, 2025.

En la interpretación del fenómeno central *”Experimentar la solicitud de retirada de la medida cautelar de urgencia”*, se considera como *condición* la categoría: “Contextualizar las consecuencias de la formalización de la denuncia”; el componente *acciones-interacciones* se verificó en las categorías: “Reconociendo la dinámica cíclica de la violencia y sus condicionantes” y “Justificando la revocación de la medida cautelar de urgencia”. Se definió como *consecuencia* la categoría: “Buscando caminos para darle un nuevo sentido a la vida”.

En este contexto, se pudo constatar que, tras la realización de la denuncia y la solicitud de la medida cautelar de urgencia, las mujeres experimentan repercusiones en sus propias vidas, en las de sus hijos y en la del agresor, lo que provoca una mezcla de emociones, además de aflorar dificultades y necesidades que deben afrontar, lo que no siempre es posible dados los recursos emocionales y sociales de que disponen.

A pesar de la condición de solicitar la retirada de la medida cautelar, reconocen la existencia de la violencia manifestada de diferentes formas, con especial énfasis en la agresión física, la violencia psicológica y moral, así como la reiteración implacable de estos episodios. Además, las motivaciones enumeradas por estas mujeres para los eventos violentos se refieren a la demanda de separación, interrelacionada con el uso de sustancias psicoactivas, como el alcohol y/o las drogas ilícitas. Sin embargo, en este movimiento de acción e interacción, buscan justificar la solicitud de revocación de la medida cautelar de urgencia, sugiriendo una oscilación entre el deseo de protección y el pesar por sus decisiones. Como consecuencia, se constata la intencionalidad de superar esta condición mediante la búsqueda de la independencia del agresor y el apego a la religiosidad.

Las interpretaciones del fenómeno también se basan en las observaciones de los profesionales de la Policía Judicial, que destacan la gravedad progresiva de los episodios de violencia y el uso de la medida cautelar como estrategia de protección y, contradictoriamente, de poder y control sobre el agresor.

El análisis permitió elaborar el modelo paradigmático presentado (Figura 1) a partir de la Teoría Fundamentada en los Datos. Esta construcción surge de la interpretación del fenómeno central—“Experimentar la solicitud de retirada de la medida cautelar de urgencia”—como un proceso experiencial cíclico y dinámico de toma de decisiones, influenciado y transversalizado por dimensiones sociales, emocionales y contextuales.

**Figura 1:** Fenómeno “Experimentando la solicitud de retirada de la medida cautelar de urgencia”. Marília, SP, Brasil, 2025.

### Condición: Contextualización de las Consecuencias de la Formalización de la Denuncia

La presentación del boletín de denuncia y la solicitud de medida cautelar de urgencia tiene consecuencias para la vida de estas mujeres, de sus hijos y también de los propios agresores. Los hijos, aunque desean la protección de sus madres, viven la ausencia del padre y sufren por ello. Los agresores se aprovechan del deseo de volver a la convivencia familiar, expresado y relatado por las mujeres en sus peticiones de reconciliación, del miedo a la cárcel y del impacto negativo en su relación laboral. Para las mujeres, las denuncias policiales provocaron impactos emocionales descritos por diversos sentimientos, como culpa, arrepentimiento, miedo, frustración y vergüenza por haber presentado la denuncia y haber iniciado el proceso judicial. Para los profesionales de la comisaría, aumenta la atención debido a los riesgos de coacción para la revocación de las MCU.

*[...] mi pequeña tuvo fiebre esta semana y me preguntó: mamá, ¿cuándo va a volver papá?* (M4)

*[...] estamos bien, pero muy molestos por lo que pasó y con miedo de que lo arresten.* (M11)

*[...] se desesperó, enviaba un montón de mensajes pidiendo perdón para volver a casa [...]* (M17)

*Por el hecho de tener que venir aquí a presentar la denuncia, me sentí muy avergonzada ante la gente.* (M13)

*[...] cuando vuelven para retirar la medida, entiendo que se han arrepentido, que muchas veces han perdonado. [...] Creo que se ven coaccionadas a solicitar la retirada, pero generalmente alegan que han actuado impulsivamente y que han perdonado.* (D3)

*[...] existe la preocupación de saber si realmente no está siendo coaccionada [...]* (D6)

### Acción-Interacción: Reconocer la Dinámica Cíclica de la Violencia y Sus Condicionantes

A pesar de solicitar la revocación de la medida cautelar, las entrevistadas reconocen que han sufrido diferentes tipos de violencia. La violencia física se manifestó en actos como puñetazos, patadas, palizas y estrangulamientos, dejando patente la maldad y crueldad del acto. La violencia patrimonial se manifestó de distintas formas y se caracterizó por la destrucción de pertenencias y bienes, lo que provocó pérdidas económicas. La violencia psicológica y moral se manifestó en las palabras del agresor, cargadas de amenazas y chantajes emocionales, términos ofensivos, desvalorizantes y despectivos, incluso con respecto a la apariencia física.

Los relatos destacan la dinámica de la convivencia conyugal basada en la recurrencia de la violencia y el incumplimiento de medidas judiciales anteriores. Para los profesionales de la Policía Judicial, existe la sensación de una gravedad progresiva de los actos y un aumento de los casos con patrones de repetición.

Él me torcía el brazo así, con tanta maldad, con tanta crueldad, que se veía en su mirada que realmente quería romperlo. (M6)

*Luego, siguió llamándome vagabunda... estás gorda y fea [...] si lo dejara, me mataría.* (M1)

*[...] no se pone agresivo conmigo, pero lanza objetos dentro de la casa.* (M15)

*Porque había una medida cautelar, pero él nunca la respetó [...]* (M19)

*[...] porque lo que era un insulto, pasó a ser una bofetada, una amenaza, me rompió el brazo, me rompió un diente. Nosotras decimos: “¿Lo siguiente será una denuncia por su muerte?”. Pero ellas no se inmutan.* (D2)

*[...] la violencia doméstica rara vez mejora, lo normal es que empeore.*(D6)

*De hecho, acaba de decir que a principios de año había solicitado una orden de protección y la revocó porque creyó en las promesas de su esposo.* (D7)

Las mujeres destacan los factores que desencadenan la violencia, incluyendo comportamientos como los celos, la posesividad, el control sobre la víctima, la falta de control emocional, la desconfianza, así como la solicitud de separación. Hay relatos de experiencias de violencia a lo largo de la vida, tanto por parte de las mujeres como de los agresores, incluyendo experiencias de relaciones abusivas anteriores y traumas infantiles. Asocian fuertemente el consumo abusivo de alcohol y drogas ilícitas con la violencia, por parte del agresor o de ambos, lo que resulta en pérdida de control y comportamientos agresivos.

*[...] me amenazó con que si me separaba o pedía el divorcio, haría de mi vida un infierno, iría a mi trabajo, me quitaría el coche [...]* (M12)

*Es celoso, muy conflictivo [...] idas y venidas, peleas, muy posesivo, veía cosas [...]* (M10)

*[...] cuando bebe, tiene un lado machista, mal genio, dice: soy malo, no soy bueno.* (M5)

*[...] tiene antecedentes de violencia [...]* M6

*Cada caso es diferente [...] en muchos casos, la víctima viene de una infancia así, para ella es normal.* (D8)

En este contexto, se considera que la motivación para acudir a la comisaría fue la búsqueda de alivio a la situación.

*[...] entonces me armé de valor y vine a la comisaría.* (M4)

*[...] para mí, la comisaría fue un punto de alivio.* (M1)

*[...] ¡vuelves a ser útil! Un ejemplo, una instrucción, decirle a la persona lo que puede hacer para mejorar esa situación [...] Es una experiencia que llevaré conmigo toda mi vida.* (D7)

### Acción-Interacción: Justificación de la Revocación de la Medida Cautelar de Urgencia

A pesar de reconocer la brutalidad de la violencia y que los factores inherentes a ella a menudo comprenden hábitos que difícilmente se eliminarán, las mujeres entrevistadas justifican la revocación por la creencia de que la violencia no se repetirá. Hay un intento de rehacer la imagen del agresor, ya sea admitiendo la culpa, alegando precipitación e incluso alegando que mintieron en el momento de la denuncia. Además, existe el deseo de mantener la familia, el arrepentimiento del agresor, los sentimientos de amor y la dependencia emocional y económica.

Los profesionales de la Policía Judicial, por su parte, indican que el uso de la medida cautelar representa una forma de ejercer poder y control sobre las acciones del agresor. Además, el desconocimiento de la Ley María da Penha lleva a utilizar la MCU como un recurso de protección temporal y de fácil revocación.

*No volverá a pasar [...] él dejó muy claro que no quería hacerlo. Pidió perdón [...]* (M2)

*Me precipité porque no era necesario [...] estamos bien, vamos a la iglesia, si algún día no funciona, me separo y listo.*(M12)

*[...] el vínculo emocional es muy fuerte.* (D4)

*Tengo que decir la verdad, mentí aun a riesgo de ser procesada [...]* (M11)

*[...] a veces es la única forma que tienen ante lo que viven, de tener poder, el control en sus manos, una moneda de cambio.* (D2)

*[...] se convence de que la persona va a cambiar porque quiere convencerse, porque le gusta la persona, entonces cree que tomar la medida y luego retirarla va a resolverlo, y no, no lo resuelve.* (D4)

### Consecuencias: Buscando Formas de Darle un Nuevo Sentido a la Vida

Las entrevistadas revelan el deseo de reconstruir sus vidas, con o sin la presencia del agresor, motivadas por la búsqueda de la independencia económica y alternativas legales, como la tramitación de procesos civiles de separación. Todavía hay quienes movilizaron recursos emocionales y espirituales para continuar con su pareja, reconociendo su propio potencial, la necesidad de diálogo y el alejamiento de familiares “potencialmente perjudiciales”.

Los profesionales de la Policía Judicial indican la necesidad de una red de atención eficaz en casos de violencia doméstica, con el fin de reducir las revictimizaciones y la revocación de las medidas cautelares.

*Quiero trabajar, entregar mi currículum. [...] Tengo un abogado y voy a separarme.* (M1)

*[...] quiero volver a trabajar, esté con él o no.* (M4)

*Empecé a hacer los cursos que quería hacer, a vivir mi vida [...] a pensar de otra manera, a ver que puedo hacer cosas sin él.* (M3)

*[...] estamos hablando mucho, yendo a la iglesia y respetándonos mutuamente.* (M12)

*[...] creo que Dios va a cambiar su forma de ser [...] eso es lo que me ha dado fuerzas, mi fe.* (M17)

*[...] realmente necesitan tratamiento psicológico [...]* (D2)

*[...] se necesita un equipo multidisciplinario para dar la primera acogida [...] profesionales de la salud, asistencia social y la defensa pública, eso sería lo necesario.* (D4)

## DISCUSIÓN

Entre las mujeres en situación de violencia doméstica de este estudio, los datos socioeconómicos, como el color de piel, el rango de edad y el nivel educativo, revelan características de pleno desarrollo reproductivo y potencial de ascenso económico y social, lo que, paradójicamente, refuerza las condiciones de vulnerabilidad de estas mujeres. En este contexto, los procesos de búsqueda de ayuda en los ámbitos jurídico y policial están impregnados de intensos sentimientos y sufrimientos, además de expresiones progresivas y cíclicas de violencia^(16)^.

Es necesario reflexionar sobre la compleja interacción entre las normas socioeconómicas y el género, ya que desafiar los elementos estructurales de la sociedad que inciden en la violencia y moldean los roles de género puede desencadenar otros factores estresantes. Estos, a su vez, aparecen en las justificaciones presentadas por las mujeres entrevistadas, con el riesgo de reducir toda la complejidad inherente al rol de género esperado en la sociedad. Además, desde la perspectiva de la masculinidad hegemónica, se mantendría el *statu quo* social y, una vez más, se culparía a la mujer^(8,9)^.

Esta comprensión favorece los debates sobre la importancia de promover aspectos protectores y emancipadores, como la ampliación de los derechos y la seguridad económica, la autonomía, un nivel educativo adecuado y una atención sanitaria eficaz, retomando el potencial de las consultas de enfermería en la APS para detectar, asesorar e intervenir en situaciones de VPI^(12)^.

En la interpretación de los datos de las entrevistas que revelan el fenómeno “Experimentar la solicitud de retirada de la medida cautelar de urgencia”, los profesionales de la comisaría alegaron que, a diferencia de otras violaciones de derechos que atendían, la violencia doméstica contra las mujeres sufre una gran influencia emocional. Este factor se convierte en una barrera para romper la relación, ya sea por la aceptación de las disculpas y las muestras de arrepentimiento, ya sea por barreras sociales relacionadas con la convivencia, las dificultades económicas y la responsabilidad sobre los hijos^(17)^.

En la misma línea de la presente investigación, estudios realizados en Estados Unidos sobre la revocación de órdenes de protección identificaron que, al formalizar las solicitudes de estas órdenes, las mujeres expresaban temor y, posteriormente, incorporaban conceptos de amor romántico a la reconciliación y al potencial de transformación. Este proceso culminaba en un esfuerzo retórico por reconstruir la imagen del agresor, valorando la paternidad y las promesas de cambio de la pareja: el ciclo de la violencia^(18,19)^.

Por lo tanto, las razones para la revocación de las medidas, al igual que en esta investigación, trascienden la mera percepción de dependencia económica de las parejas íntimas, ya que implican factores multidimensionales^(10)^. Estos factores justifican la contradicción de sentimientos que impregnan el proceso de revocación, relatados por las mujeres involucradas en este fenómeno. Este hecho demuestra que las decisiones conscientes de mantener la relación se basan, a menudo, en la creencia de que afrontar las posibles consecuencias de salir de la relación podría empeorar la situación, como un comportamiento adaptativo derivado de distorsiones cognitivas^(20,21)^, lo que configura un ciclo vivido por estas mujeres y confirmado por los profesionales de la comisaría.

La condición de ausencia de contacto impuesta por la medida cautelar de urgencia, en los términos de la ley, genera la sensación de perjuicio a la paternidad del agresor. Por ello, los procesos judiciales que involucran a niños requieren mayor atención para garantizar la seguridad y el bienestar del niño y de la madre, que en su día fue víctima^(17)^. La creencia de que las necesidades de los hijos están por encima de las suyas propias lleva a estas mujeres a permanecer en la relación, incluso si eso significa exponerse al riesgo de revictimización^(17,18)^.

Aunque solicitaron la revocación de la MCU, la interpretación de los datos revela que reconocen la existencia de diferentes tipos de violencia, incluida la agresión física, a menudo caracterizada por actos brutales con lesiones físicas y emocionales duraderas, lo que aumenta los impactos sobre la salud mental, como la depresión y la ansiedad^(22)^. El reconocimiento de la violencia psicológica y moral, por su parte, se presenta como la tipificación de violencia más dolorosa e insidiosa, con repercusiones que repercuten en la autoestima y la salud mental, lo que da lugar a síntomas psíquicos y trastorno de estrés postraumático. Se configura como un sistema sutil y asfixiante de control y poder, marcado por la sumisión y el miedo, que sumerge a estas mujeres en una espiral de angustia y vulnerabilidad^(23)^.

Además, en los relatos recopilados se observa una dinámica familiar violenta, de carácter cíclico y progresivo, que afecta a la capacidad de la mujer para establecer conexiones y dificulta la solicitud de ayuda. Además, este patrón repercute en otras relaciones e interacciones sociales de la familia, debilitando el papel parental^(24)^. Estos hallazgos remiten a los supuestos del interaccionismo simbólico, que postula que los seres humanos actúan en el mundo según los significados atribuidos en las interacciones sociales, moldeados por su capacidad comunicativa^(14)^.

Además, el alcohol y otras sustancias psicoactivas están fuertemente asociados con la violencia doméstica contra las mujeres. Los patrones de violencia perpetrados por hombres ebrios son impredecibles e ilógicos, lo que desencadena una percepción errática de culpa en las víctimas, que no pueden prever ni evitar tales actos^(25)^. En este contexto, las condiciones de vida y las relaciones sociales orientan la percepción de los individuos en su forma de vivir^(14)^.

Aunque los resultados apuntan al celo masculino como factor desencadenante de conflictos y violencia, el celo femenino también puede desempeñar ese papel. Sin embargo, existe una justificación socialmente aceptable de que el celo masculino sería una acción reactiva al comportamiento de la mujer, una culpabilización^(26)^. Esta idea, derivada de la masculinidad hegemónica, refuerza los estereotipos y estigmas que rodean el tema de la violencia contra las mujeres.

Los factores anteriormente discutidos y vinculados a la historia personal de los agresores, influenciados por perspectivas socioculturales y familiares, pueden ser una variable explicativa de la violencia doméstica en la edad adulta, con implicaciones para las futuras prácticas parentales^(27)^.

Las emociones desempeñan un papel mediador de significados, especialmente cuando se considera el IS. El miedo, la culpa y el apego se reinterpretan dentro de la dinámica de la relación, y la permanencia en una situación de riesgo cobra sentido para las mujeres en situación de violencia, reforzando el apego emocional a los agresores. La percepción de los matices emocionales y psicológicos de estas mujeres, junto con las estrategias de apoyo, es fundamental para una asistencia de enfermería que tenga como objetivo romper este ciclo perverso^(14,23,24)^.

Los recursos de la asistencia sanitaria deben combinarse con la aplicación de la ley, con el fin de proporcionar a la sociedad la atención debida, ya que la violencia doméstica constituye una violación de los derechos humanos, en consonancia con los principios globales de erradicación de la violencia contra las mujeres^(1,2)^. En este sentido, es necesario un enfoque que tenga en cuenta no solo los aspectos legales, sino también los significados y las dinámicas simbólicas que sustentan estas actitudes^(14)^.

La percepción del riesgo que supone una relación violenta motiva a las mujeres a buscar ayuda, lo que puede justificarse por la escalada de la violencia, el deseo de proteger a los hijos, la ruptura de la intergeneracionalidad de la violencia, el sentimiento de miedo por su propia vida y/o la de sus hijos, y los cambios en la percepción del vínculo romántico con el agresor, factores que coinciden con los resultados del presente estudio^(28)^. Este proceso destaca que los significados se transforman según la interpretación que la persona hace de los acontecimientos de su vida cotidiana, siempre inmersos en las relaciones sociales^(14)^.

El valor de la mujer en situación de violencia al enfrentarse al sistema legal refleja el deseo de poner fin a la espiral de violencia, haciendo pública la violación de sus derechos. La tipificación específica de la violencia doméstica en los códigos legales de varios países refleja la necesidad de responsabilizar a los agresores, contrarrestando la cultura de impunidad y garantizando la protección de las víctimas^(29)^. Para garantizar esta protección, la Ley María da Penha, en Brasil, prevé la instauración de medidas cautelares de urgencia, a través de la solicitud de las víctimas, lo que desencadena la activación de los demás mecanismos judiciales al recurrir a la Comisaría de Policía Judicial^(10)^.

El silencio de muchas mujeres y la revocación de las medidas cautelares reflejan un patrón de supervivencia que no debe interpretarse como pasividad. Por el contrario, debe entenderse como una respuesta a condiciones de alta vulnerabilidad, moldeadas por múltiples factores, como los estilos de comunicación, las estrategias de autorregulación emocional, las habilidades para la resolución de conflictos y la percepción de los hechos. La decisión de la mujer de mantener la relación abusiva puede verse como un intento de lidiar con la incertidumbre y los riesgos asociados, especialmente cuando el apoyo institucional y social es limitado ^(20)^. A la luz del interaccionismo simbólico, este comportamiento está moldeado por los significados que estas mujeres atribuyen a sus experiencias^(14)^.

Muchas mujeres experimentan una disonancia cognitiva ante los primeros signos de violencia, oscilando entre negar el peligro y justificar el comportamiento^(20)^. Este juicio es un proceso mental profundamente influenciado por las interacciones simbólicas^(14)^, como los mensajes recibidos de su contexto social, especialmente del agresor y de los hijos.

Los equívocos y los prejuicios perpetúan las percepciones estereotipadas, subyugando la autonomía de las mujeres en momentos críticos en los que podrían ejercerla. El apoyo sistemático y formal ha demostrado ser dos veces más eficaz para salir de las relaciones violentas en los dos años siguientes, en comparación con aquellas que no recibieron estos servicios^(30)^. Además, no se han descrito otros tipos de violencia, lo que refuerza la idea de la naturalización de este fenómeno en la sociedad, especialmente en relación con las masculinidades y feminidades tradicionales que refuerzan los comportamientos estereotipados^(8,9)^.

Es necesario un cambio estructural y cultural sobre cómo se percibe la violencia, a menudo oculta por dinámicas emocionales complejas. Se debe invertir en estrategias preventivas y de apoyo que capaciten a las mujeres para recuperar su autonomía y seguridad, favoreciendo el protagonismo de la Enfermería de Atención Primaria en el acompañamiento de las mujeres en situación de violencia.

### Limitaciones Del Estudio

Se considera un factor limitante del estudio la especificidad del enfoque, que se centró en las mujeres en situación de violencia que acudieron a la comisaría para solicitar la revocación de la medida cautelar de urgencia. Otros aspectos del complejo universo de las mujeres en situación de violencia limitan la generalización de los resultados, como aquellas que abandonaron los procesos judiciales remitidos al Foro, las que no revocaron formalmente la medida cautelar y reinterpretaron sus experiencias, así como las víctimas de feminicidio.

### Contribuciones Al Ámbito de la ENfermería, la Salud o Las Políticas Públicas

El estudio contribuye con un enfoque integral sobre el tema de la violencia doméstica contra las mujeres, destacando el potencial de los profesionales de la salud, en especial de la enfermería en la APS, que en sus prácticas comunicativas pueden ayudar a estas mujeres a reinterpretar sus vidas. Se refuerza la perspectiva de los derechos de género, promoviendo la autonomía de las mujeres en la toma de decisiones y la protección contra la revictimización, un papel esencial de la enfermería en la búsqueda de la erradicación de esta violencia. Se pretende contribuir al campo del proceso de cuidado, desde una perspectiva interdisciplinaria, junto con políticas actualizadas de aplicación de la ley para un enfoque libre de estereotipos que tienden a subyugar la autonomía.

## CONSIDERACIONES FINALES

En la interpretación de las experiencias de las mujeres en situación de violencia doméstica que solicitaron la revocación de las medidas cautelares de urgencia, se evidencia la complejidad y la magnitud de los retos a los que se enfrentan. Al optar por retirar las medidas, evalúan las repercusiones de la decisión no solo sobre ellas mismas, sino también sobre sus hijos y el agresor, y se ven atravesadas por sentimientos ambiguos, como culpa, arrepentimiento y tristeza, incluso cuando reconocen la experiencia violenta y los factores que contribuyen a su continuidad.

Las dificultades para comprender y sostener el proceso judicial penal, asociadas a la persistencia del ideal del amor romántico y al vínculo parental con los agresores, constituyen motivos frecuentes para la revocación de las medidas cautelares. En muchos casos, las mujeres se resisten a romper completamente con la dinámica familiar violenta, creyendo que su mantenimiento garantiza algún tipo de estabilidad emocional o material a los hijos o al núcleo familiar.

Sin embargo, surgen de manera significativa estrategias para reinterpretar sus vidas, lo que revela que, incluso ante un escenario adverso y decisiones complejas, estas mujeres demuestran intencionalidad en la reconstrucción de sus trayectorias. Este proceso no ocurre de manera lineal o inmediata, sino que se manifiesta en la búsqueda de la autonomía, el fortalecimiento de la fe y la construcción de nuevas perspectivas de vida, lo que indica movimientos de superación y resistencia.

Cabe destacar que los referentes teóricos adoptados—IS y TFD—fueron oportunos para la interpretación de las experiencias de las mujeres en situación de violencia doméstica, permitiendo visibilizar las dinámicas simbólicas y emocionales que influyen en sus decisiones y percepciones. Se espera que nuevos estudios en la materia puedan contribuir a la calificación de la atención, promoviendo intervenciones sensibles y eficaces que permitan romper el ciclo de violencia y construir nuevas posibilidades sociales para las mujeres.

## Data Availability

El conjunto de datos relativos a los resultados trabajados en el software NVIVO Plus, versión 11, está disponible en: https://doi.org/10.48331/SCIELODATA.RRXQOX.
